# CRISPR-Switch regulates sgRNA activity by Cre recombination for sequential editing of two loci

**DOI:** 10.1038/s41467-019-13403-y

**Published:** 2019-11-29

**Authors:** Krzysztof Chylinski, Maria Hubmann, Ruth E. Hanna, Connor Yanchus, Georg Michlits, Esther C. H. Uijttewaal, John Doench, Daniel Schramek, Ulrich Elling

**Affiliations:** 1 Vienna Biocenter Core Facilities, Vienna Biocenter (VBC), Dr. Bohr Gasse 3, Vienna, Austria; 2 Institute of Molecular Biotechnology of the Austrian Academy of Science (IMBA), Vienna Biocenter (VBC), Dr. Bohr Gasse 3, 1030 Vienna, Austria; 3 Broad Institute of MIT and Harvard, Cambridge, MA USA; 4 Centre for Molecular and Systems Biology, Lunenfeld-Tanenbaum Research Institute, Mount Sinai Hospital, Toronto, ON Canada; 5 Department of Molecular Genetics, University of Toronto, Toronto, ON Canada

**Keywords:** Cell growth, CRISPR-Cas9 genome editing

## Abstract

CRISPR-Cas9 is an efficient and versatile tool for genome engineering in many species. However, inducible CRISPR-Cas9 editing systems that regulate Cas9 activity or sgRNA expression often suffer from significant limitations, including reduced editing capacity, off-target effects, or leaky expression. Here, we develop a precisely controlled sgRNA expression cassette that can be combined with widely-used Cre systems, termed CRISPR-Switch (SgRNA With Induction/Termination by Cre Homologous recombination). Switch-ON facilitates controlled, rapid induction of sgRNA activity. In turn, Switch-OFF-mediated termination of editing improves generation of heterozygous genotypes and can limit off-target effects. Furthermore, we design sequential CRISPR-Switch-based editing of two loci in a strictly programmable manner and determined the order of mutagenic events that leads to development of glioblastoma in mice. Thus, CRISPR-Switch substantially increases the versatility of gene editing through precise and rapid switching ON or OFF sgRNA activity, as well as switching OVER to secondary sgRNAs.

## Abstract (web summary)

Inducible genome editing systems often suffer from leakiness or reduced activity. Here the authors develop CRISPR-Switch, a Cre recombinase ON/OFF-controlled sgRNA cassette that allows consecutive editing of two loci.

## Introduction

CRISPR-Cas9 systems provide highly efficient genome editing tools using engineered single guide RNAs (sgRNA) and a simple and robust RNA-DNA hybridization-based target recognition^1–4^. Some CRISPR-Cas9 applications, such as conditional gene disruption, phenotyping of essential genes, or sgRNA library screening require strict spatial and/or temporal control. Thus, several inducible systems regulating the expression of both Cas9 and sgRNA have been developed^5–8^. For example, modified or split Cas9 enzymes can be activated by light or small molecules^7,9–17^. Although the activity of such systems can be precisely controlled, either spatially or temporally, they frequently suffer from substantially reduced DNA-editing efficiency^7,9–12^. Doxycycline (DOX)-inducible transcription of Cas9 can be regulated without significant reduction in editing activity, but such systems often show substantial leakiness, leading to premature editing and necessitating clone preselection^13–17^. Methods for regulating sgRNA expression are mostly based on DOX-inducible Pol III transcription of sgRNAs, which equally suffer from leakiness or reduced editing efficiency^18–20^. Moreover, none of these systems provides a method for sequential editing of two loci.

Here, we describe a switchable ON/OFF sgRNA expression system using a modified, loxP-containing sgRNA architecture, which we term CRISPR-Switch (SgRNA With Induction/Termination by Cre Homologous recombination). We show that CRISPR-Switch can be rapidly induced and terminated, being highly active in the ON-state and fully repressed in the OFF-state. Benchmarking to alternative systems confirmed the superior performance of CRISPR-Switch. We also present a two-step strictly ordered editing methodology, allowing sequential induction of mutagenic events at two loci, and use it to analyze the order of mutations that trigger tumorigenesis in vivo.

## Results

### **C**ontrol of sgRNA activity using Cre-Lox/Flp-FRT systems

**Fig. 1:**
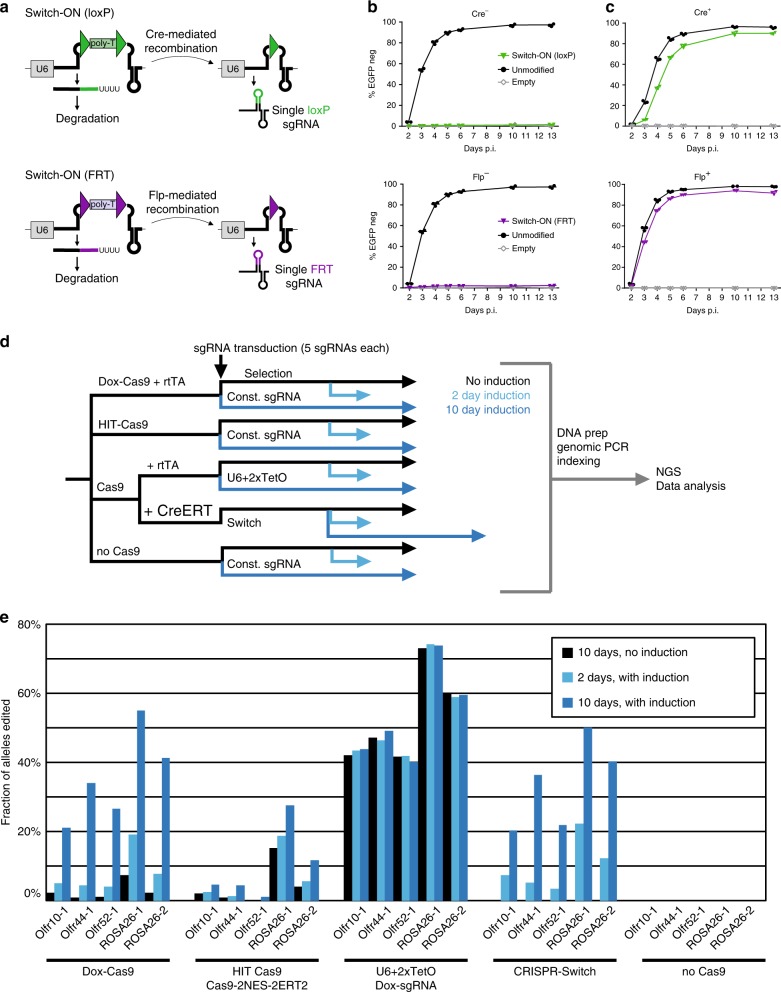
Recombinase-based inducible sgRNAs. **a** Locus architecture of sgRNA induction systems in “off” (left) and “on” (right) states switchable via recombination. sgRNA and sgRNA-coding sequence is shown as thick line stem-loop structure and recombination sites are represented as triangles. U6 – U6 promoter, poly-T – polythymidine termination signal. **b, c** Recombinase-inducible sgEGFP1 guide RNAs were introduced into EGFP and Cas9-expressing mES cells without (**b**) or with (**c**) constitutive recombinase expression and EGFP loss was monitored with flow cytometry over 13 days post infection (p. i.). **d** Benchmarking experimental setup. Mouse embryonic stem cells were transduced with Cas9, HIT Cas9, or DOX-inducible Cas9 plus rtTA and selected for successful infection. Subsequently, cells expressing constitutive Cas9 were transduced with rtTA-GFP or CreERT2-GFP and sorted for expression by flow cytometry. All resultant cell lines were separately infected with sgRNA constructs targeting five non-essential loci in the mouse genome and transduced cells were selected. Induction was for 0, 2, or 10 days, respectively, to quantify leakiness and activity of respective constructs. Upon DNA lysis of all samples, PCR amplification, and next generation sequencing (NGS), resulting indels were mapped. **e** Relative activity of different sgRNAs was reproducible across systems and days. Source data are provided as a Source Data file.

We chose to develop a fully active/repressed switchable system for Pol III transcribed sgRNA based on site-specific Cre and Flp recombinases. Cre and Flp harbor very high recombination efficiencies in many cell types, can be precisely controlled both spatially and temporally and are commonly used to switch-on or abrogate expression of RNA polymerase II transcribed protein-coding genes (Supplementary Fig. 1a)^21^. However, sgRNAs are short RNA polymerase III transcripts that lack introns and untranslated (UTR) regions, precluding the insertion of recombination sites in these features. To engineer the Switch-ON cassettes we identified three sites within the sgRNA expression cassette where both a poly-T STOP signal could block expression, and the recombination site remaining after STOP excision might be tolerated for sgRNA activity: (i) within the U6 promoter regulatory elements, where the STOP cassette would block promoter activity by altering the distance between the TATA-box and proximal sequence element (PSE), which has been previously shown to block shRNA expression^22^ (Supplementary Fig. 1b); (ii) at the 5’-end of the sgRNA-coding sequence, which would cause premature termination of sgRNA transcription (Supplementary Fig. 1c), similar to the BLADE approach^23^; or (iii) at the apex of the sgRNA repeat:anti-repeat stem-loop inspired by other modifications in this region as e.g. in the SAM system^24^, which would terminate transcription within the scaffold sequence, removing part of the sgRNA essential for Cas9 binding (Fig. 1a, Supplementary Fig. 1d).

To systematically assess the activity and regulation of the three STOP signal-containing sgRNA cassettes, we compared them to control sgRNA scaffolds, including the standard scaffold and the extended, optimized sgRNA scaffold^25^, all targeting EGFP (sgEGFP1). Mouse embryonic stem cells (mES cells) harboring a homozygous EGFP expression cassette and constitutively expressed *S. pyogenes* Cas9 were used to test the efficiency of the poly-T STOP cassettes. In the absence of Cre, none of the tested constructs showed significant EGFP loss compared to the control (0.9 ± 0.1–1.6 ± 0.6%, vs 0.3 ± 0.3% in control, ± refers to s.d., Fig. 1b, Supplementary Fig. 2a, b). In an mES cell line that expressed Cre recombinase (EGFP+ Cas9+ Cre+), all constructs showed efficient EGFP loss (Fig. 1c, Supplementary Fig. 2c). The Switch-ON construct with a loxP insertion within the sgRNA scaffold ((iii) above) resulted in the fastest and most efficient EGFP mutagenesis (96 ± 0.8% vs 80 ± 1.4–82 ± 1% for both U6- and 5’-located STOP cassettes). Similarly, an FRT-based construct also showed efficient induction of EGFP mutagenesis without overt signs of leakiness (Fig. 1a–c). In absence of Cre/Flp, neither loxP nor FRT interfere with sgRNA activity when compared to conventional control sgRNA constructs (Supplementary Fig. 2d). Of note, however, in the mES cell lines expressing Cre, loxP-STOP-loxP resulted in a slight delay in EGFP loss. A similar delay occurred when the sgRNA contained a single loxP in a Cre+, but not in a Cre− cell line, suggesting a general effect of Cre interfering with transcription of loxP-containing loci (Fig. 1c and Supplementary Fig. 2e). The delay, though, did not affect the overall efficiency. In FRT-based constructs we did not observe such delay in editing in the presence of Flippase (Fig. 1b, c). To determine if recombination sites are tolerated in the context of other sgRNAs, we cloned an additional six sgRNAs targeting EGFP into the same set of vectors and analyzed the kinetics of EGFP loss by flow cytometry (Supplementary Fig. 3). While minor kinetic differences were observed, possibly due to multiplicity of infection, overall the scaffolds containing single loxP and FRT outperformed the standard scaffold and showed similar kinetics to the optimized sgRNA scaffold.

We tested the inducibility and tightness of our constructs in an mES cell line harboring CreERT2 (EGFP+ Cas9+ CreERT2+). Cre recombinase activity was induced immediately or 7 days post infection (p. i.) using 4-OH tamoxifen (Supplementary Fig. 3b), resulting in robust EGFP deletion, with the STOP cassette within the scaffold again showing the highest activity (91.8 ± 7.6% deletion efficiency). The rate of EGFP loss was comparable at induction on day 0 and day 7. A very low level of leakiness observed in the CreERT2 line likely results from a leaky cytoplasmic retention of CreERT2 or spontaneous recombination during virus production and can be further reduced by use of modified STOP cassettes carrying floxed antibiotic resistance genes (Supplementary Fig. 4a). To corroborate these findings with an alternative readout, we targeted five non-essential loci in a Cas9+, CreERT2+ cell line with inducible sgRNAs selecting for viral integration by blasticidin resistance located between loxP sites. Indeed, leakiness of our inducible sgRNA system appeared non-detectable with NGS, yet the construct is inducible to high efficiency (Supplementary Fig. 4b, c)

*Staphylococcus aureus* Cas9 is widely used due to its smaller size, enabling it to be packaged in adeno-associated virus (AAV) for in vivo delivery^26^. To test whether loxP/FRT Switch-ON constructs would be tolerated in *S. aureus* sgRNAs and to test the system on an endogenous human locus, we designed *S. aureus* Flp- and Cre-inducible sgRNA scaffolds targeting CD81, a cell surface marker. We transduced them into human melanoma (A375) cells and monitored CD81 loss (Supplementary Fig. 5). Again we observed highly efficient and precisely controlled editing activity.

We concluded that the scaffold placement of loxP-STOP-loxP between the repeat and anti-repeat sequence of sgRNAs is an ideal setup to control generation of active sgRNAs for CRISPR-Cas9 systems. Given the high activity as well as tightness of scaffold-located STOP cassettes, we focused on this setup and termed the system CRISPR-Switch for SgRNA With Induction/Termination by Cre Homologous recombination.

### CRISPR-Switch-ON enables sharp induction of editing

We leveraged the inducibility of CRISPR-Switch-ON to knockout essential genes by integration and antibiotic selection of sgRNA cassettes in the OFF-state. The system was tested on 16 essential genes by designing 4 sgRNAs per gene and infecting EGFP-positive mES cells carrying inducible Cre (EGFP+ CreERT2 + Cas9+). Mouse ES cells infected with virus encoding mCherry and constitutive sgEGFP1 to generate mCherry+, EGFP− cells were used as internal control. These cells were mixed with cells carrying inducible sgRNAs against the essential genes, subsequently loxP recombination and editing was induced by 4-OH tamoxifen. The population was monitored for 10 days post induction by flow cytometry (Supplementary Fig. 6). We measured depletion of sgRNA-containing, EGFP+, mCherry− cells relative to EGFP−, mCherry+ controls. For 15 of 16 genes and most sgRNAs, we observed an over 10-fold depletion of EGFP+ cells, corresponding to >90% efficiency in generating loss-of-function alleles upon induction of editing (Supplementary Fig. 6). Thus, CRISPR-Switch allows to uncouple sgRNA transduction and selection from subsequent rapid and robust induction of sgRNAs and gene editing, thus enabling the study of transient and time-resolved phenotypes.

### **B**enchmarking of CRISPR-Switch to alternative systems

A multitude of inducible editing systems have been described^5–7,9–18^, we therefore aimed to compare our system to published alternatives with regards to leakiness in the OFF-state and activity in the ON-state. To this end, we chose systems demonstrated to be highly active and tight, based on previous benchmarking experiments^8,14,27^. Cas9 and all required genetic elements were introduced into mouse ES cells polyclonally (Fig. 1d), selected for, and subsequently sgRNAs targeting five endogenous loci were transduced. On-target loci were analyzed by PCR followed by next generation sequencing (NGS) at three timepoints, namely after 2 and 10 days with induction as well as after 10 days without induction. DOX-Cas9, HIT-Cas9, and U6 + 2xTetO all showed activity in the induced state and relative activity of different sgRNAs correlated well between the systems. However, substantial leakiness in the OFF-state was also observed in all cases^8,14,27^. In particular for the DOX-inducible sgRNA system (U6 + 2xTetO), leakiness was indistinguishable from the induced state in our specific setup. Despite an extended selection protocol for CRISPR-Switch, possibly resulting in some silencing of Cas9 or CreERT2, activity of CRISPR-Switch reached comparable levels in the ON-state, yet again no leakiness was observed without induction (Fig. 1e). This was in good alignment to the results in a clonal cell line, where even much higher activity but no leakiness was observed (Supplementary Fig. 4b, c). Taken together, conditional editing by CRISPR-Switch outperforms alternative systems on endogenous loci as it represents the only tight conditional regimen tested.

### Time-limited sgRNA expression using CRISPR-Switch-OFF

**Fig. 2:**
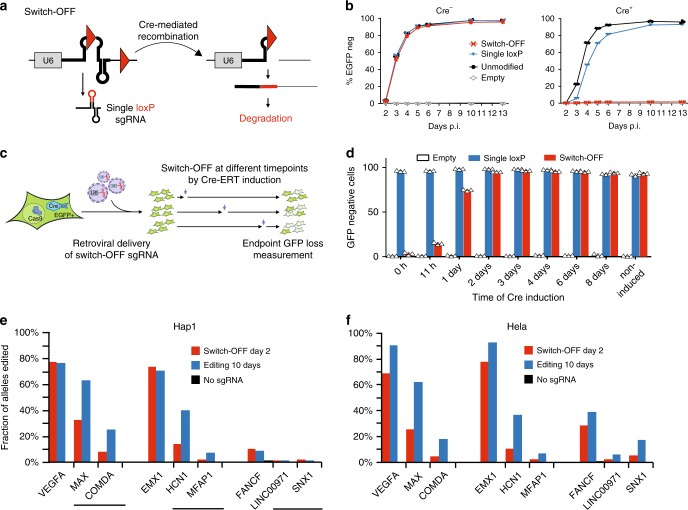
Inducible Switch-OFF of sgRNA expression. **a** Locus architecture of sgRNA Switch-OFF system in “on” (left) and “off” (right) states. sgRNA and sgRNA-coding sequence is shown as thick line stem-loop structure and recombination sites are represented as triangles. Single loxP-derived stem-loop within sgRNA scaffold produced in the “on” state is shown in red. U6 – U6 promoter. **b** EGFP loss kinetics (see Fig. 1b, c) obtained by Switch-OFF sgRNA in absence (left) and presence (right) of Cre. sgRNA constructs with optimized scaffold and no targeting sequence or with sgEGFP1 were used as negative and positive controls, respectively. Error bars represent standard deviation (*n* = 3). **c** Experimental setup. Cassettes encoding sgEGFP1 are delivered on retroviruses into EGFP and Cas9 encoding cells. Switch-OFF is induced at different time-points post infection and the level of EGFP loss is measured for all the induction time-points 14 days p. i. **d** EGFP loss obtained after gEGFP1 Switch-OFF at different time-points post infection in red. Constitutively expressed single loxP sgEGFP1 was used as a positive control. Error bars represent standard deviation (*n* = 3). Cas9+ Hap1 cells (**e**) and Hela cells (**f**) targeted with sgRNAs show high editing rates on target (bold, VEGFA, EMX1, FANCF), but also for respective off-target sites (MAX, COMDA; HCN1, MFAP1; LINC00971, SNX1;), when editing continuous for ten days (blue), while termination of editing by Cre transduction two days subsequent to sgRNA transduction (red) halts editing. Termination of editing results in a better on-/off-target ratio due to near-saturation of on-target editing already at day 2. Source data are provided as a Source Data file.

The ability to precisely terminate CRISPR-Cas9 activity also has several applications, including designing synthetic gene circuits by reversibly modulating gene expression using CRISPRi (inhibition) or CRISPRa (activation), as well as improving the ratio of on-target to off-target cutting. To assess the feasibility of terminating sgRNA activity, we generated a CRISPR-Switch-OFF construct. Placement of one loxP site at the apex of repeat:anti-repeat stem-loop and a second one downstream of the sgRNA termination sequence (Fig. 2a) will result in the removal of essential 3’ section of the sgRNA upon recombination, rendering the sgRNA inactive. Importantly, this design retains the guide sequence information for subsequent identification of targeted cells in screening paradigms. To test this design, we introduced sgEGFP1 in the CRISPR-Switch-OFF setup into mES cells (EGFP+ Cas9+) with or without Cre recombinase expression. In the absence of Cre, the sgRNA was highly active, providing rapid deletion of EGFP (Fig. 2b). In the Cre-expressing line almost no EGFP deletion was detected (1.4 ± 0.2% EGFP− cells on day 6; 1.5 ± 0.05% and 1.5 ± 0.2% EGFP− cells on days 10 and 13 p. i.), suggesting Switch-OFF efficiently abrogates sgRNA activity. We introduced the same construct into the mES cells expressing CreERT2 (EGFP+ CreERT2+ Cas9+), induced Switch-OFF at different timepoints after infection and measured EGFP deletion 10 days p.i. (Fig. 2c, d). Induction of Switch-OFF at the time of infection and 11 h p.i. resulted in low rates of deletion (3.5 ± 1.5% and 14.8 ± 1.3%, respectively), confirming rapid and efficient Switch-OFF. By 2 days p.i. EGFP deletion was already saturated (Fig. 2d). We thus speculated that beyond on-target saturation, off-target editing will continue at lower kinetics and negatively affecting the on-/off-target ratio of sgRNAs.

To test if limiting editing activity by CRISPR-Switch-OFF can be used to reduce off-target editing, we transduced two human cell lines, namely Hap1 and Hela, with three sgRNAs each, that were reported to have strong off-target activities^28,29^. Editing was either continued for 10 days or terminated 2 days subsequent to sgRNA transduction by additional transduction with Cre recombinase. On- and off-target loci were analyzed by NGS on DNA obtained at day 10. Similar to targeting EGFP in mES cells, we observed near completion of on-target editing for highly active sgRNAs targeting VEGF and EMX1 at day 2 (Fig. 2e, f). In contrast, however, off-target editing followed slower kinetics and consequently continued to increase until day 10. Thus, termination of editing improved on-/off-target ratios by a factor of 0.9–3.9 in Hap1 and 1.9–3.1 in Hela cells. In summary, Switch-OFF can terminate editing rapidly and efficiently while maintaining information about the sgRNA target sequence to improve on-/off-target activity.

**Fig. 3:**
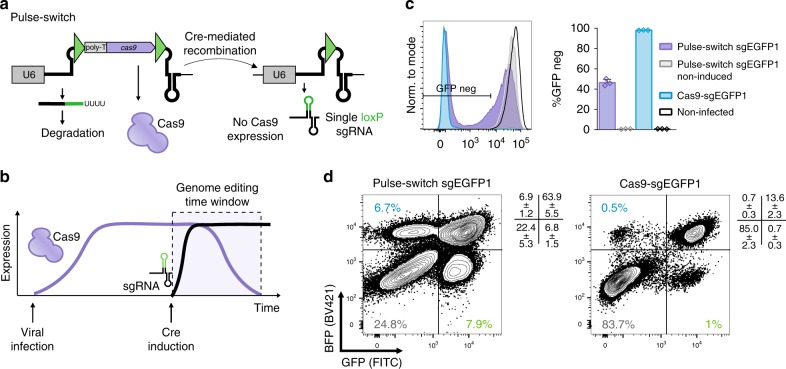
CRISPR-Switch-Pulse. **a**, **b** Schematics of CRISPR pulse construct. The *Cas9* cassette provides expression in a non-recombined state (**a**, left) while sgRNA transcription is terminated by polythymidine signal (poly-T). Upon recombination the *Cas9* cassette is removed and sgRNA is transcribed (**a**, right) leading to a short time window of both sgRNA and Cas9 present in a cell (**b**). U6 – U6 promoter. Triangles represent recombination sites. **c** EGFP deletion efficiency of CRISPR-Switch-Pulse with sgEGFP1 and constitutively expressed Cas9 with sgEGFP1 was compared in CreERT2, EGFP-positive mES cells. Error bars represent standard deviation (*n* = 3). **d** Switch-Pulse and constitutive Cas9 lentivirus with sgEGFP1 guide RNA targeting both EGFP and EBFP2 was introduced to NIH3T3 cells encoding CreERT2, EGFP, and EBFP2. Efficiency of single and double deletion was assessed with flow cytometry in FITC and BV421 channels. Plus refers to s.d.

Limited gene editing can also be used to intentionally and consistently generate incomplete editing saturation and thus heterozygous genotypes. To further reduce the temporal window of editing, we inserted Cas9 under control of the EF1α promoter into the loxP-STOP-loxP cassette of a Switch-ON construct (Switch-Pulse, Fig. 3a, b). In this setup, Cas9 and sgRNA expression are mutually exclusive: Cas9 is initially transcribed and the sgRNA is not. Cre recombination excises and eliminates Cas9 while reconstituting an active sgRNA. This generates a short editing window defined by the kinetics of Cas9 mRNA and protein stability and induction of sgRNA expression. We targeted EGFP and EBFP2 transgenes as a detectable proxy representing two alleles in NIH3T3 cells and monitored loss of one or both alleles by flow cytometry. 13.8 ± 0.4% of cells displayed heterozygous outcomes, a 10-fold enrichment compared to standard lentiviral vector encoded Cas9 and sgEGFP1 (Fig. 3c, d). Thus, intentionally limited saturation editing can be seamlessly integrated into CRISPR-based screening pipelines.

### Consecutive gene editing of 2 loci by CRISPR-Switch-OVER

**Fig. 4:**
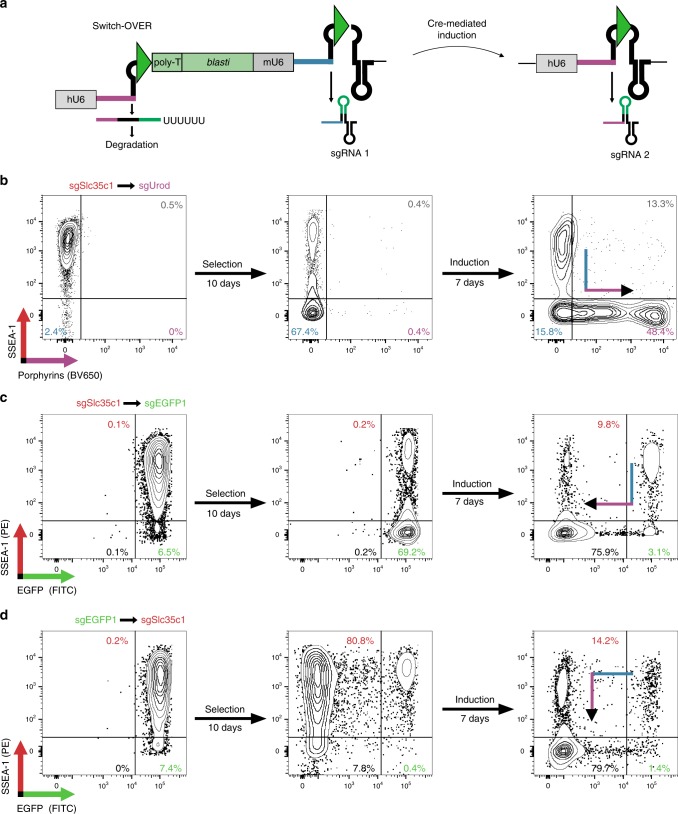
CRISPR-Switch OVER allows consecutive editing on two loci. **a** Construct design. Two sgRNAs are encoded under control of their own RNA pol III promoters (hU6, human U6 RNA promoter; mU6, mouse U6 RNA promoter). Non-induced state (left): active sgRNA1 (blue) is transcribed, sgRNA2 (purple) is prematurely terminated by a poly-T STOP cassette. Green triangles denote recombination sites. Induced state (right): recombination removes STOP cassette, mU6 promoter, and sgRNA1 guide sequence, allowing expression of active sgRNA2. Antibiotic resistance cassette (*blasti*) counter selects for premature recombination. **b** Consecutive deletion of Slc35c1 and Urod in mES cells. Slc35c1 deletion leads to down-regulation of SSEA-1 as determined by flow cytometry. Recombination activates sgRNA against *Urod*, leading to accumulation of fluorescent porphyrins. Plots show representative result of a triplicate. **c**, **d** Sequential deletion of *Slc35c1* resulting in SSEA-1 loss followed by EGFP editing (**c**) or vice versa (**d**) illustrating tight directionality of the approach.

In many cellular processes, two or more signaling events are involved. CRISPR editing can be used to interfere with or generate such signals genetically; however, editing of two loci in a defined temporal order requires two different Cas9 enzymes and sgRNA systems or repeated delivery. To simplify sequential editing, we combined the CRISPR-Switch ON and OFF systems to create CRISPR-Switch-OVER, in which Cre recombination switches the essential part of the scaffold from one sgRNA to another, rendering the first sgRNA inactive while activating the second sgRNA (Fig. 4a).

To test the system in mES cells, we generated constructs that would switch from an sgRNA targeting *Slc35c1* to an sgRNA targeting *Urod*. Loss of Slc35c1 will result in loss of the mES cell surface marker SSEA-1, loss of Urod to subsequent accumulation of protoporphyrin IX and thus will result in increased autofluorescence of cells. Cells transduced with the construct lost the SSEA-1 epitope, but remained autofluorescence low. Upon activation of CreERT2 cells gained autofluorescence through the loss of Urod (Fig. 4b and Supplementary Fig. 7). Similar results were obtained for constructs that targeted *Slc35c1* and *EGFP*, with the loss of Slc35c1 followed by loss of EGFP, or vice versa (Fig. 4c, d, Supplementary Fig. 8).

Sequential editing in vivo is particularly useful for modeling tumorigenesis, where several driver mutations synergize to produce cellular transformation. However, reinfection of the same cells in vivo with viral constructs is often very challenging or impossible due to low infection efficiency. We thus tested if CRISPR-Switch-OVER can be leveraged in vivo to study mutational synergy in a scenario of clonal tumorigenesis with few transduced cells within the tissue. Mutations in NF1 and TP53 are found in 20 and 34% of glioblastoma multiforme (GBM) patients, respectively, and those two mutations show significant co-occurrence in ~10% of GBM patients (p-value 0.002) (Supplementary Fig. 9). Inactivation of *Trp53* and *Nf1* in mice results in glioma development^30,31^ and the order of their inactivation is thought to influence tumorigenicity^32^, since Nf1 loss is thought to lead to a Trp53-dependent apoptosis or senescence. For tumors to form, *Trp53* thus has to be inactivated before or concomitantly with *Nf1* loss. We therefore tested the ability of the CRISPR-Switch-OVER system to determine the sequential path of tumorigenesis in a mouse model of *Nf1*/*Trp53* mutant glioma.

**Fig. 5:**
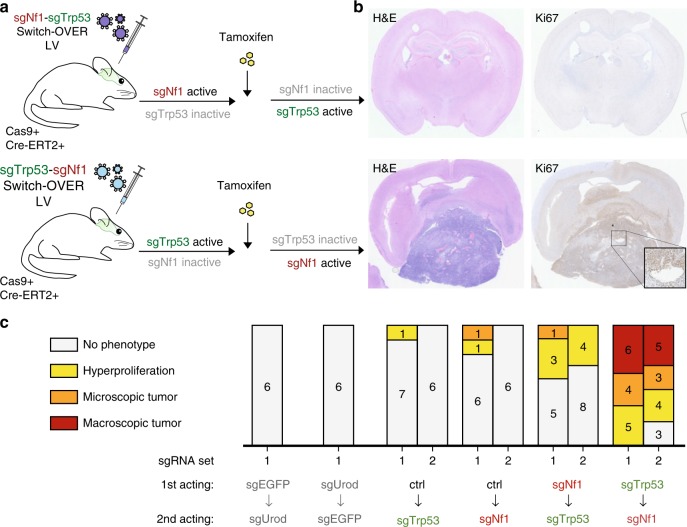
Recombinase-mediated consecutive editing in vivo. **a** In vivo *Nf1*/*Trp53* mutant GBM mouse model. Schematic of experimental setup. Lentiviral particles encoding Switch-OVER constructs were injected into the right ventricle of Cas9-GFP; Nestin-CreERT2 mice at postnatal day (P) 0 with indicated dual sgRNA lentivirus. At P21, mice were treated with tamoxifen and followed for 8-10 months. **b** Representative images showing coronal full brain section stained with H&E as well as anti-Ki67 antibody. (**c**) Stacked bar graph shows frequency of tumors and hyperproliferation in 8-month-old mice (numbers correspond to number of animals) transduced with the indicated dual sgRNA lentiviral constructs. Except for *Urod*/*EGFP* combinations, two different sets of sgRNA sequences were used as indicated.

Upon confirming in vivo activity of our constructs using the Urod paradigm (Supplementary Fig. 10a), we generated constructs that either first target *Trp53* and then *Nf1* after recombination, or contrariwise. Mice with constitutive Cas9 and Nestin-CreERT2 expression were infected by stereotactic injection with Switch-OVER lentivirus. After 3 weeks, tamoxifen administration was used to switch editing from the first locus to the second locus. All brains were analyzed for tumors after 8-10 months (Fig. 5a, b and Supplementary Fig. 10b). While >50% of mice in which *Trp53* was edited before *Nf1* came down with tumors with 37% harboring macroscopic tumors, no tumor development was observed in mice in which *Nf1* was edited before *Trp53*, and only one control mouse with an sgRNA targeting *Nf1* presented one microscopic (Trp53 negative) tumor nodule (Fig. 5c). These data indicate that in mouse, Trp53 loss prior to Nf1 loss promotes tumorigenesis, while Nf1 loss in a Trp53-proficient cell is not tolerated, thus preventing the generation of tumorigenic cells mutated in both genes. Moreover, these data confirm that CRISPR-Switch-OVER is functional for consecutive editing of two genes in vivo.

## Discussion

We present CRISPR-Switch as an efficient and flexible system to achieve conditional editing through control of sgRNA activity. The system is not detectably leaky in the OFF-state, provides highly efficient genome editing in the ON-state, allows ordered editing of two loci, and functions both in vitro and in vivo. Paired with the many available Cre- and CreERT2-expressing cell lines and mouse models, CRISPR-Switch enables a variety of possible applications for both temporal and spatial control of editing.

Induction of editing by modulating sgRNA activity is a viable approach for studying phenotypes in temporal or special manner, but also of essential genes, as it decouples selection for sgRNA-expressing cells from onset of editing. Several inducible editing systems have been reported till date, controlling activity either on the side of Cas9 or sgRNA. For example, efficient control of CRISPR-Cas9 editing via Cre recombinase and loxP-STOP-loxP has been reported using the conditional expression of Cas9^33^. However, controlling CRISPR-Cas9 editing through Cas9 expression has several disadvantages. First, even in the absence of sgRNAs, Cas9 expression can affect cell fitness^34^, possibly due to spurious nuclease activity or a general effect on transcription, whereas no adverse effects of sgRNA expression have been reported in absence of Cas9. Second, induction of Cas9 in vivo may result in an immune response^35–37^, which is problematic for tumor models^38^. Thus, cells lacking Cas9 are not an appropriate control for many experimental setups. In some approaches Cas9 is converted into an inactive split protein that can be activated by small molecules, light, or the presence of an sgRNA^7,9–12^. These approaches could circumvent Cas9 immunogenicity but are characterized by lowered editing activity (typically >40% reduction)^7,9–12^. CRISPR-Switch avoids these pitfalls by controlling editing through sgRNA activity. Previous systems for inducible expression of sgRNAs, based on DOX-inducible promoters or small molecules, often suffer from leakiness in the OFF-state, low sgRNA expression levels upon induction or diminished editing activity^18–20,39^. Cre-based activation of sgRNAs was used previously in a transient transfection-based assay to generate logic gates of transgene activity^23^. However, this system, similar to our 5’ loxP variant (Supplementary Figure 1c), was not tested for gene-editing of endogenous loci. We systematically benchmarked CRISPR-Switch and found CRISPR-Switch to be the only fully tight system, while activity in the ON-state reached comparable levels.

In turn, limiting editing activity is reported to reduce off-target activity^11,16,40,41^. Indeed, we show that termination of editing improved on-/off-target ratio up to 3.9-fold. Importantly, CRISPR-Switch-OFF retains the specific sgRNA targeting sequence, required e.g. for pooled screens. We further show that the mutually exclusive, but temporally overlapping expression of first Cas9 and then an sgRNA can, based on expression and stability kinetics, generate a short editing window, which results in the efficient generation of heterozygous genotypes.

CRISPRa and CRISPRi approaches require continuous targeting to the locus and thus maintained expression of the sgRNA^42,43^. CRISPR-Switch-OFF is therefore expected to also allow for reversible modulation of gene expression in CRISPRa/i experiments e.g. for counterselection in screens or temporal gene activation/repression in differentiation regimen. We expect that future experiments will combine CRISPR-Switch with an ever-growing number of CRISPR systems and applications based on intrinsic challenges of various assays.

CRISPR-Switch-OVER represents a simple setup for efficient consecutive expression of different sgRNAs, which enables sequential locus editing. Importantly, this system neither requires integration of two independent Cas systems into the experimental regimen nor repeated delivery of sgRNAs. In addition, it strictly excludes an unwanted order of editing by its design. We have used this system in vitro and in vivo to investigate the required order of mutagenic events in glioma tumorigenesis, finding that loss of Trp53 must precede loss of Nf1. We expect that the research community will find ample use in various paradigms, such as sequential editing or overexpression of factors to induce cell type conversions. Further, consecutive editing may be superior to the simultaneous expression of two sgRNAs at once, as it avoids competition for Cas9 binding of sgRNAs with different affinities and offers a valuable control population. Thus, CRISPR-Switch will also facilitate screening with libraries targeting e.g. putatively synthetic genes or paralogues.

## Methods

### Molecular cloning

Cloning strategies of individual constructs and primer sequences used are given in Supplementary Tables 1, 2 and 5.

For introduction of fragments via PCR and for mutagenesis, plasmid DNA was amplified using 25 ng of template, 2.5 µM phosphorylated oligos, 3% DMSO and Phusion High Fidelity MM (Thermo). Cycling conditions were 1 × 98 °C 1 min; 30 × 98 °C 15 s, 72 °C 3.5 min; 1 × 72 °C 10 min. PCR product was purified over column (Gel purification kit, Thermo) followed by treatment with DpnI FD (Thermo) as per manufacturer’s instructions. 100 ng column purified DNA was incubated with T4 DNA ligase (NEB) in a standard ligation reaction. *E. coli* STBL3 were used for DNA transformation. Oligonucleotide phosphorylation was done using 300 pm of oligo with 10 U T4 PNK (NEB), 1 x T4 DNA ligase buffer (NEB) in final volume of 50 µl at 37 °C for 30 min. Excess of ATP was removed by GE25 column (GE healthcare).

For sgRNA cloning (see Supplementary Table 3 for sgRNA targeting sequences) and oligo cloning, the plasmid backbone was digested with BsmBI HF (NEB), BpiI FD (Thermo), or BamHI FD (Thermo) and EcoRV FD (Thermo) (see Supplementary Table 1) following manufacturer’s instructions and purified over column (Gel purification kit, Thermo). Excess of ATP was removed by GE25 column (GE healthcare). Oligos were mixed to the concentration 8.3 µM in 33 mM NaCl and annealed by denaturation and subsequent slow cooling (95 °C 10 min, −10 °C/min). 80 ng of linearized plasmid was ligated with 0.5 µM annealed oligos in final volume of 10 µl using 5 U T4 DNA ligase (Thermo).

Gibson assembly 2 x master mix (NEB) was used as recommended by manufacturer for all the Gibson assembly-based clonings.

Cloning strategy of Switch-OVER sgRNA constructs is outlined in Supplementary Fig. 11. In brief: oligonucleotides encoding two sgRNA sequences (only targeting sequence; rc- reverse-complement of targeting sequence) together with cloning overhangs (capital letters) and BbsI (for retrovirus) or BsmBI (for lentivirus) cloning sites (small letters, recognition site underlined) were designed (RetroF: CACC-sgRNA2-GTTTgggtcttcgagaagacctTTTG-sgRNA1, RetroR: AAAC-sgRNA1rc-CAAAaggtcttctcgaagacccAAAC-sgRNA2rc; LentiF: CACC-sgRNA2-GTTTggagacggacgtctctTTTG-sgRNA1, LentiR: AAAC-sgRNA1rc-CAAAagagacgtccgtctccAAAC-sgRNA2rc) and cloned into appropriate single loxP construct. Subsequently, the resulting vector was opened with BbsI/BsmBI and insert cassette (Switch-OVER insert: EF1a-Blasti For in vitro experiments and EF1a-Puro for in vivo experiments) was ligated in.

### Cell culture

Mouse ES cells (feeder-free) were grown at 37 °C in 5% CO_2_ in ES cell medium (ESCM): 450 ml DMEM (Sigma–Aldrich), 75 ml FCS (Invitrogen), 5.5 ml penicillin–streptomycin (Sigma–Aldrich), 5.5 ml NEAA (Sigma–Aldrich), 5.5 ml L-Glutamine (Sigma–Aldrich), 5.5 ml NaPyr (Sigma–Aldrich), 0.55 ml βME stock solution (70 µl pure βME, Sigma M7522, in 20 ml PBS), and 10^3^U ESGRO/ml (Millipore). For standard maintenance, cells were trypsinized (Trypsin, Gibco) every second day and replated. Packaging cells (Platinum-E (Cell Biolabs) Retroviral Packaging Cell Line and LentiX (Clontech) lentiviral packaging cell line) for producing Retrovirus and Lentivirus were cultured at 37 °C in 5% CO_2_ in DMEM (Sigma–Aldrich) supplemented with 15% FBS (Invitrogen), 1% penicillin–streptomycin (Sigma–Aldrich), 1 mM NaPyr (Sigma–Aldrich) and 4 mm L-Glutamine (Sigma–Aldrich). NIH 3T3 mouse embryonic fibroblasts were grown 37 °C in 5% CO_2_ in DMEM (Sigma–Aldrich) supplemented with 10% FBS (Gibco), 2 mM L-Glutamine (Sigma–Aldrich) and Normocin (Invivogen). A375 cells were obtained from the Cancer Cell Line Encyclopedia and were maintained at 37 °C in 5% CO_2_ in RPMI 1640 (VWR International) supplemented with 10% FBS (Invitrogen). HEK293T cells used for the production of all *S. aureus* sgRNA virus were obtained from ATCC (CRL-3216). HEK293T cells were cultured at 37 °C in 5% CO_2_ in DMEM (VWR International) supplemented with 10% FBS (Invitrogen).

### Cell lines used

Embryonic stem cells (AN3-12 cells) are a clonal derivative of HMSc2, mouse embryonic stem cells derived in the laboratory (Elling et al. 2011 and 2017); NIH3T3 cells were purchased from ATCC, PlatinumE cells were purchased from Cell Biolabs, and LentiX cells were purchased from Clontech. A375 cells were obtained from Cancer Cell Line Encyclopedia. Hap1 cells were obtained from Haplogen and Hela cells from ATCC. All cell lines were confirmed to be mycoplasma negative regularly.

### Virus production

Retroviral constructs were introduced into Platinum-E retroviral packaging cells (Cell Biolabs) by calcium phosphate transfection. In brief, 20 µg of transfer plasmid were mixed with 125 µl 1 M CaCl_2_ and diluted to 500 µl. The plasmid mixture was added dropwise to 500 µl 2x HBS under agitation, incubated for 15 min at room temperature for precipitate formation and added to Platinum-E cells (50% confluency in a 10 cm dish). Medium was changed after 16–20 h and virus was harvested 16 h later. When needed, a second harvest was done after 8 h. Before infection, virus containing supernatant was filtered through 0.45 µm syringe filters.

Lentiviral constructs (Switch-Pulse) were introduced into LentiX (Clontech) lentiviral packaging cells by polyethylenimine (PEI)-mediated transfection. In brief, 6 µg of transfer plasmid, 3 µg of gag-pol packaging helper plasmid and 1.5 µg of VSV-G envelope helper plasmid were mixed in 1 ml DMEM followed by addition of 33 µl of 1 mg/ml PEI (Polysciences Inc.) and vortexing. After 15 min incubation at room temperature the mixture was added to LentiX cells (50% confluency in a 10 cm dish). Medium was changed after 16–20 h and virus was harvested 16 h later. When needed, second harvest was done after 8 h. Before infection, virus containing supernatant was filtered through 0.45 µm syringe filters.

All virus used for *S. aureus* sgRNA testing was produced using the TransIT-LT1 (Mirus) transfection reagent according to the manufacturer’s protocol. HEK293T cells were seeded 24 h prior to transfection in 6-well dishes at a density of 1.5 × 10^6^ cells per well in 2 ml of DMEM + 10% FBS. On the day of transfection, one solution of Opti-MEM (Corning, 66.25 μl) and LT1 (8.75 μl) was combined with a DNA mixture of the packaging plasmid pCMV_VSVG (Addgene 8454, 250 ng), psPAX2 (Addgene 12260, 1250 ng), and the transfer vector (1250 ng). This solution was incubated at room temperature for 20–30 min and media was gently changed on the HEK293T cells. After the incubation, the transfection mixture was added dropwise to the HEK293T cells; the plates were then centrifuged at 1000 × *g* for 30 min at room temperature. Following centrifugation, plates were transferred to a 37 °C incubator for 6–8 h, after which the media was removed and gently replaced with fresh media (DMEM + 10% FBS supplemented with 1% BSA).

For in vivo infections, lentiviral particles were produced as follows: HEK293NT (Invitrogen R700-07) cells were seeded onto poly-L-lysine coated 15 cm^2^ plates (12 plates/construct) and transfected with the lentiviral Switch-OVER constructs and lentivirus packing plasmids pMD2 and pPAX2 (Addgene plasmid 12260 and 12259) using polyethyleneimine (Cedarlane 23966-1) and serum free media (Wisent 319-015-CL) for 8 h. After transfection 293 s were then grown in media supplemented with 10% FBS (Wisent 080-150) and 1% penicillin/streptomycin (Wisent 450-201-EL) and grown for 48 h. Three hundred and sixty milliliter of supernatant was harvested and filtered through Stericup-HV PVDF 0.45-μm filter (Millipore SCHVU02RE) and ultracentrifugation with a sucrose cushion in an mlS-50 rotor (Beckman Coulter) at 100 000 g for 1.5 h. Viral pellet was resuspended in 60 µl PBS. Viral concentration was determined through infecting R26-LSL-tdtomato mouse embryonic fibroblasts and using flow cytometry to measure viral activated fluorescent expression.

### Switch-ON leakiness assessment

mES cells (Cas9+, EGFP+) were infected with retroviruses encoding sgEGFP1 with the Switch-ON scaffold or with Switch-ON scaffold modified by introduction of blasticidin resistance into loxP-STOP-loxP cassette (lox-Blasti-lox). sgEGFP1 with single loxP scaffold and empty sgRNA constructs with both Switch-ON scaffolds were used as positive and negative controls, respectively. 1 day post infection (p.i.) antibiotic selection was started, with neomycin (Gibco, 0.5 mg/ml) used for Switch-ON and blasticidin (Gibco, 5 µg/ml final concentration) for modified Switch-ON constructs. The cells were kept under antibiotic selection for the whole duration of the experiment and monitored for EGFP loss with flow cytometry (BD Fortessa) for 17 days.

### Kinetics of EGFP deletion in mES cells

To test EGFP deletion efficiency provided by sgRNAs with diverse scaffolds, constructs encoding sgRNAs with seven different EGFP targeting sequences (sgEGFP1-7) in combination with four different scaffolds (standard, optimized, single loxP and single FRT) were prepared and used together with corresponding empty controls to prepare retroviral particles. mES cells (Cas9+, EGFP+) were infected with all sgRNA combinations in 24-well format. 1 day p.i. neomycin selection was started (Gibco, 0.5 mg/ml) and cells were monitored for EGFP loss with flow cytometry (BD Fortessa) as indicated.

To test tightness and activity of diverse Switch-ON constructs, Cre-negative (Cas9+, EGFP+) and Cre-positive (Cre+, Cas9+, EGFP+) mES cells were infected in 96-well format with retroviruses encoding sgEGFP1 in Switch-ON, U6-Switch-ON and 5’-Switch-ON setups as well as retroviruses encoding sgEGFP1 with optimized scaffold and empty sgRNA construct with optimized scaffold used as positive and negative control, respectively. Flippase-negative (Cas9+, EGFP+) and Flippase-positive (Flp+, Cas9+, EGFP+) mES cells were infected in 96-well format with retroviruses encoding sgEGFP1 in Switch-ON (FRT) setup as well as retroviruses encoding sgEGFP1 with optimized scaffold and empty sgRNA construct with optimized scaffold used as positive and negative control, respectively. 1 day p.i. neomycin selection was started (Gibco, 0.25 mg/ml) and cells were monitored for EGFP loss with flow cytometry (BD Fortessa) for 13 days.

For testing deletion kinetics with diverse Switch-ON constructs, inducible Cre (CreERT2, Cas9+, EGFP+) mES cells were infected in 96-well format with retroviruses encoding sgEGFP1 in Switch-ON, U6-Switch-ON and 5’-Switch-ON as well as retroviruses encoding sgEGFP1 with optimized scaffold and empty sgRNA construct with optimized scaffold used as positive and negative control, respectively. Two sets of plates were prepared, one set was left non-induced and in the second set, CreERT2 was induced at the day of infection (day 0) with 4OH-tamoxifen (Sigma–Aldrich, 0.5 µM final concentration), added to the culture for following 7 days. 1 day p.i. neomycin selection was started (Gibco, 0.25 mg/ml). On day 6 p.i., non-induced cultures were split into two sets, one set was left non-induced and in the second set, CreERT2 was induced at the day 7 p.i. with 4OH-tamoxifen (Sigma–Aldrich, 0.5 µM final concentration), added to the culture for following 4 days. All the cells were monitored for EGFP loss with flow cytometry (BD Fortessa) for 13 days.

### Benchmarking

Mouse embryonic stem cells were transduced with lentiviral constructs carrying Cas9-P2A-Puro (a kind gift from Lukas Dow, Addgene #110837), HIT Cas9 (EFa-Cas9-2xNES-2ERT2, a kind gift from Yu Wang, Addgene #120551), or rtTA and TetO-Cas9 (pCW-Cas9, a kind gift from Eric Lander and David Sabatini, Addgene #50661). Subsequent to selection, cells carrying Cas9-P2A-puro were infected with retroviral constructs carrying CreERT2 or rtTA and GFP as selection marker. Upon establishment of all polyclonal cell lines, cells were infected with respective sgRNA constructs (see Supplementary Table 4, constitutive sgRNAs in improved backbone, in U62xTetO, or in CRISPR-Switch) in 6-well format and selection was performed. For all conditions but CRISPR-Switch, infection and selection was performed in presence and absence of relevand inducing agent. For CRISPR-Switch, selection was only performed in absence of 4OH-tamoxifen, given that the selection cassette is lost upon recombination. This might have resulted in some silencing of Cas9, CreERT, or the sgRNA construct and thus could have -if any- only a negative impact on activity relative to the other conditions. DNA samples were harvested as indicated in Fig. 2 and processed for PCR by DNA lysis (Lysis buffer: 0.5% N-Laurylsarcosine, 10 mM Tris, 10 mM EDTA, 10 mM NaCl, adjusted to pH 8 with HCl, 1 mg/ml Proteinase K (VWR)), 10 ul lysate was diluted with 90ul water and proteinase K heat inactivated at 95 °C for 10 min. Of this solution aliquots of 16 ul were used as template input for 50ul PCR reactions using a touchdown PCR protocol (95 °C 3 min, [95 °C 20 sec, 65 °C 20 sec with an increment of −0.3 °C per cycle, 72 °C 30 sec,] × 23 cycles, [95 °C 20 sec, 58 °C 20 sec, 72 °C 30 sec,] × 30cycles, 72 °C 3 min). Primers are listed in Supplementary Table 5. Experimental indices were added by a secondary PCR step of 10 cycles with dual indexing (Illumina). Products are pooled and sequenced on a MySeq in a SR150 run. Sequence read mapping, indel calling and quantitative kinetic analysis is carried out with custom python scripts deposited on Github (https://github.com/GMichlits/VBC-Score) in folder Scripts/Fig 3. In brief, NGS reads are mapped based on identification of pimer Sequences present, indels determined with a custom algorithm and quantified based on read number. Indel annotation used is mutation_type:size:position, e.g. d:4:-2 annotates a deletion of 4 bp starting 2 bp upstream of the predicted CRISPR cut site between bases 17 and 18 of a 20-nt sgRNA.

### Off target analysis

Hap1 and Hela cells were transduced with a lentiviral construct carrying Cas9-P2A-Puro (a kind gift from Lukas Dow, Addgene #110837). SgRNAs targeting VEGFA, EMX, and FANCF (Supplementary Table 6) were cloned into the Switch-OFF vector, packaged in amphotropic retroviral particles using PlatA cells (Cell Biolabs), and transduced in 6-well format. Selection was initiated 24 h later by G418 addition to medium. Another 24 h later, half of the cells were transduced with a lentiviral vector carrying Cre and hygro resistance (Addgene # 34565). Indel analysis was performed as for benchmarking.

### Switch-OFF in mES cells

To test Switch-OFF setup tightness in the OFF state and activity in the ON state, Cre-negative (Cas9+, EGFP+) and Cre-positive (Cre+, Cas9+, EGFP+) mES cells were infected in 96-well format with retroviruses encoding sgEGFP1 with Switch-OFF scaffold as well as retroviruses encoding sgEGFP1 with single loxP scaffold and empty sgRNA construct with single loxP scaffold used as positive and negative control, respectively. One day p.i. neomycin selection was started (Gibco, 0.25 mg/ml) and cells were monitored for EGFP loss with flow cytometry (BD Fortessa) for 13 days.

For testing kinetics and tightness of induced Switch-OFF, inducible Cre-positive mES cells (CreERT2+, Cas9+, EGFP+) were seeded in sets in 24-well format and infected with retroviruses encoding sgEGFP1 with Switch-OFF scaffold as well as retroviruses encoding sgEGFP1 with single loxP scaffold and empty sgRNA construct with single loxP scaffold used as positive and negative control, respectively. One day p.i. neomycin selection was started (Gibco, 0.5 mg/ml). In each set Cre activity was induced with 4OH-tamoxifen (Sigma–Aldrich, 0.5 µM final concentration; induction kept for 3 days) at different timepoints ranging from the time of infection (0 h) to 8 days p.i., as indicated. Non-induced set was used as an additional control. EGFP loss was measured with flow cytometry (BD Fortessa) at day 11 p.i.

### Essential sgRNAs

For control cell preparation, mES cells (CreERT2+, Cas9+, EGFP+) were infected with a retrovirus encoding sgEGFP1, neomycin resistance and mCherry. Following antibiotic selection (G418, Gibco, 0.5 mg/ml) control cells were sorted for EGFP- and mCherry + with BD FACS Aria III.

mESC (CreERT2+, Cas9+, EGFP+) were infected with retroviruses encoding sgRNAs targeting essential genes in Switch-ON cassettes in 96-well plates. One day p.i. neomycin (Gibco, 0.5 mg/ml final concentration) selection was started. Five days p.i., neomycin-selected EGFP + mES cells containing sgRNAs against essential genes or empty control sgRNA were mixed in 1:1 ratio with mCherry+/EGFP− control cells. 24 h later 4OH-tamoxifen (Sigma–Aldrich, 0.5 µM final concentration, 3 days of induction) was added to the cell mixture (day 0).

Essential gene knockout-induced cell death of EGFP + mES cells was monitored by flow cytometry (BD Fortessa) with mCherry+/EGFP− cells used to control for unspecific cell death. Experimental readout was the ratio of EGFP+ cells over mCherry+ control cells measured by flow cytometry (ratio of number of events within ‘single cells’ population) on day 0 and every day until day 10 post induction. For calculation of fold depletion, the obtained ratios were normalized to day 0.

### *S. aureus* sgRNA testing

All lentiviral transductions with A375 cells were performed as follows. Cells were seeded in 6-well plates at a density of 5.5 × 10^5^ cells per well in a final volume of 1.1 ml per well, in the presence of lentivirus and 0.5 µg/ml polybrene. Cells were incubated with virus overnight at 37 °C, after which the viral media was replaced with fresh media. 2 days p.i., cells were selected with the appropriate selection drug.

For *S. aureus* sgRNA testing, A375 cells were first transduced with Cre-hygroR or Flp-EGFP. After Cre-expressing cells had selected with hygromycin (50 μg/ml) for at least 2 weeks, parental, Cre-expressing, and Flp-expressing cells were transduced with the various *S. aureus* sgRNAs; puromycin selection (1 μg/ml) was applied 2 days p.i. and maintained for the remainder of the experiment.

To monitor CD81 protein loss, cells were collected and stained with APC-conjugated anti-CD81 antibody (Biolegend 349510) diluted 1:100 in flow buffer (PBS, 2% FBS, 5 μM EDTA) for 30 min on ice. Cells were then washed twice with flow buffer prior to assessment by flow cytometry with the BD Accuri C6 Sampler system.

### CRISPR pulse

mES cells (CreERT2+, EGFP+) were infected with lentiviruses encoding sgEGFP1 and Cas9-p2a-blasti (kind gift from Julian Jude) or sgEGFP1 Pulse-Switch construct. Cells were selected with blasticidin (Gibco, 5 µg/ml final concentration) for 4 days starting at day 1 p.i. Subsequently, the cultures were split into 2 sets, one set was kept under blasticidin selection and in the second set, CreERT2 was induced with 4OH-tamoxifen (Sigma–Aldrich, 0.5 µM final concentration) for 48 h. On day 12 p.i. EGFP loss was measured with flow cytometry (BD Fortessa).

WT NIH 3T3 mouse embryonic fibroblasts were infected with CreERT2-GFP retrovirus and subsequently with a lentivirus encoding EBFP2. Cells were further sorted for GFP and EBFP2 and expanded clonally. Resulting NIH3T3 cells (CreERT2+, GFP+, EBFP2+) were infected with lentiviruses encoding sgEGFP1 and Cas9-p2a-blasti or with sgEGFP1 Pulse-Switch construct in 24-well format. Cells were selected with blasticidin (Gibco, 5 µg/ml final concentration) for 2 days starting at day 1 p.i. Subsequently, the cultures were split into two sets of plates, one set was kept non-induced and in the second set, CreERT2 was induced with 4OH-tamoxifen (Sigma–Aldrich, 0.5 µM final concentration) for 4 days. On day 10 p.i. (day 7 post induction) GFP and EBFP2 loss was measured with flow cytometry (BD Fortessa).

### Consecutive gRNAs in vitro

For in vitro experiments, retroviral constructs with blasticidin selection cassette within the insert were used. MES cells (CreERT2+, Cas9+, EGFP+) were infected in 24-well format. Blasticidin selection (Gibco, 5 µg/ml final concentration) was started at day 1 p.i. At day 10 p.i. plates were split into two sets, one set was left non-induced and kept under blasiticidin selection and in the second set recombination was induced with 4OH-tamoxifen (Sigma–Aldrich, 0.5 µM final concentration). Induction was continued for 3 days (day 12 p.i.) and cells were cultured until day 17 p.i. SgRNA activity was assessed with flow cytometry (BD Fortessa) on day 10 and day 17 p.i. EGFP signal and protoporphyrin IX autofluorescence were measured in FITC and BV650 channels respectively. For Ssea-1 depletion measurement, cells were surface-stained with PE-coupled anti-Ssea1 antibody (anti-mouse, Biolegend). To this end, cells were collected, washed with PBS, spun down (here and after 300 × *g*, 5 min, 4 °C), resuspended in 200 µl of PBS and transferred to V-bottom 96-well plate followed by additional spinning step. Cell pellets were resuspended in 25 µl FACS buffer (1xPBS, 1 mg/ml NaN_3_, 5 mg/ml BSA, 0.5 mM EDTA) containing 0.5 µl Fc_γ_ block (eBioscience). Samples were incubated on ice for 30 min followed by diluting with FACS buffer up to 200 µl, spinning down, washing with FACS buffer and pelleting. For Ssea-1 staining, the pellets were resuspended in 25 µl FACS buffer containing 0.5 µl of PE-coupled anti-Ssea1 antibody (anti-mouse, Biolegend). Samples were incubated on ice for 30 min in the dark, followed by diluting with FACS buffer up to 200 µl, spinning down and washing with FACS buffer.

### P0 intraventricular injections

Virus was mixed with 0.05% Fastgreen (F7252-5G) before loading into a syringe (Hamilton 7659-01) with a 33-gauge needle (Hamilton 7803-05). P0 pups were anesthetized on ice. Their head was secured into position with a raised mold. A stereotactic manipulator was used to position the needle to 0.3 mm above the Bregma towards the Lambda Suture and 0.1 mm lateral of Sagittal Suture into the right ventricle. The needle was injected 3 mm into the skull and retracted 1 mm for a final depth of 2 mm. One micromicroliter of virus was injected and allowed 1 min to diffuse before retraction of the needle. Post-injection, the neonates were warmed by cupping them in hands until conscious. We have complied with all relevant ethical regulations for animal testing and research. Animal husbandry, ethical handling of mice and all animal work were carried out according to guidelines approved by Canadian Council on Animal Care and under protocols approved by the Centre for Phenogenomics Animal Care Committee (18-0272 H).

### Mouse tissue sample preparation

Mouse brains were fixed with 4% paraformaldehyde (Electron Microscopy Sciences 15710), dehydrated in an ethanol series using a Leica ASP300 automatic tissue processor and embedded in paraffin wax using Leica Histocore Arcadia h. Samples were sectioned at 4.5 µM with a Leica RM2255 semi-automatic microtome.

### Immunohistochemistry

Tissue was treated with 3% H_2_O_2_ (Fisher H325-500) in PBS (Wisent 311-012-1 L) prior to rehydration in water to remove any endogenous peroxidases. Antigen retrieval was performed using a 10 mM Na Citrate pH6 solution (Wisent 609-096-CL) in a microwave for 15 min. Primary antibody was applied at 4 °C overnight using p53 (Leica NCL-L-p53-CM5p, 1:1000) and ki67 (Abcam ab15580, 1:1000). Goat anti-rabbit secondary (Vector Labs BA-1000, 1:500) was applied for 30 min followed by ABC reagent (Vector Labs PK-6100 according to manufacturer’s specifications) for 25 min and developed with DAB (Vector Labs SK-4100) for 4 min or less. Tissue was counterstained with Harris Hematoxylin (Sigma HHS128-4) for 8 min and mounted with Shur Mount (Electron Microscopy Sciences 17991-01). Images of stained samples were obtained by using NPD.view2 software (Hamamatsu U12388-01).

### In silico modeling of RNA secondary structures

Secondary structure prediction was performed with RNAfold (http://rna.tbi.univie.ac.at/cgi-bin/RNAWebSuite/RNAfold.cgi) with manual adjustments in agreement with the published sgRNA structure. RNA structures were visualized with VARNA (http://varna.lri.fr/).

### Reporting summary

Further information on research design is available in the Nature Research Reporting Summary linked to this article.

## Supplementary information

Supplementary Information

Reporting Summary

## Data Availability

The data supporting the findings of this study are available within the paper and its supplementary information files. Sequencing data are available from NCBI BioProject under BioProject ID: PRJNA587837 and BioSample SAMN13220215. All materials are available upon request. The source data underlying Figs. 1, 2, 3, as well as Supplementary Figs 2, 3, 4, 6 are provided as a Source data file. Any other relevant data are available upon reasonable request.

## Source data

Source Data

## Code availability

Bioinformatic code used for this manuscript is available at https://github.com/GMichlits/CRISPR-Switch
