# Supplementary material for: CRISPR-Switch regulates sgRNA activity by Cre recombination for sequential editing of two loci
Source: Nat Commun. 2019 Nov 29;10:5454. doi: 10.1038/s41467-019-13403-y (PMC6884486; doi:10.1038/s41467-019-13403-y)
Supplement: Supplementary file 1 — Supplementary Information [file 41467_2019_13403_MOESM1_ESM.pdf]

Supplementary Information file to:

CRISPR-Switch regulates sgRNA activity by Cre recombination for sequential editing of two loci

Krzysztof Chylinski[1*] and Maria Hubmann[2*], Ruth E. Hanna[3], Connor Yanchus[4,5], Georg Michlits[2], Esther C. H. Uijttewaal[2], John Doench[3], Daniel Schramek[4,5], Ulrich Elling[2, #]

Content:

Supplementary Figure 1 – Recombinase-based STOP cassettes.

Supplementary Figure 2 – Alternative sgRNA activation systems.

Supplementary Figure 3 – Comparison of EGFP loss kinetics obtained by sgRNAs with different scaffolds.

Supplementary Figure 4 – Further improvement of tightness by antibiotic resistance cassette.

Supplementary Figure 5 – Recombinase-based inducible sgRNAs for *Staphylococcus aureus* CRISPR/Cas9.

Supplementary Figure 6 – targeting essential genes using CRISPR-Switch-ON

Supplementary Figure 7 – Recombinase-mediated consecutive editing *in vitro*

Supplementary Figure 8 – Recombinase-mediated consecutive editing *in vitro*

Supplementary Figure 9 – TP53 and NF1 mutations in GBM.

Supplementary Figure 10 – Recombinase-mediated consecutive editing *in vivo.*

Supplementary Figure 11 – Construction of Switch-OVER vectors.

Supplementary Table 1 – Basic plasmid constructs

Supplementary Table 2 – Synthetic DNA sequences used for backbone construction

Supplementary Table 3 – sgRNA targeting sequences

Supplementary Table 4 - Mouse ON target forward oligos for sgRNA cloning ordered

Supplementary Table 5 – Editing-scar sequencing primer

Supplementary Table 6 - Human ON/OFF target sites

Supplementary Figure 1

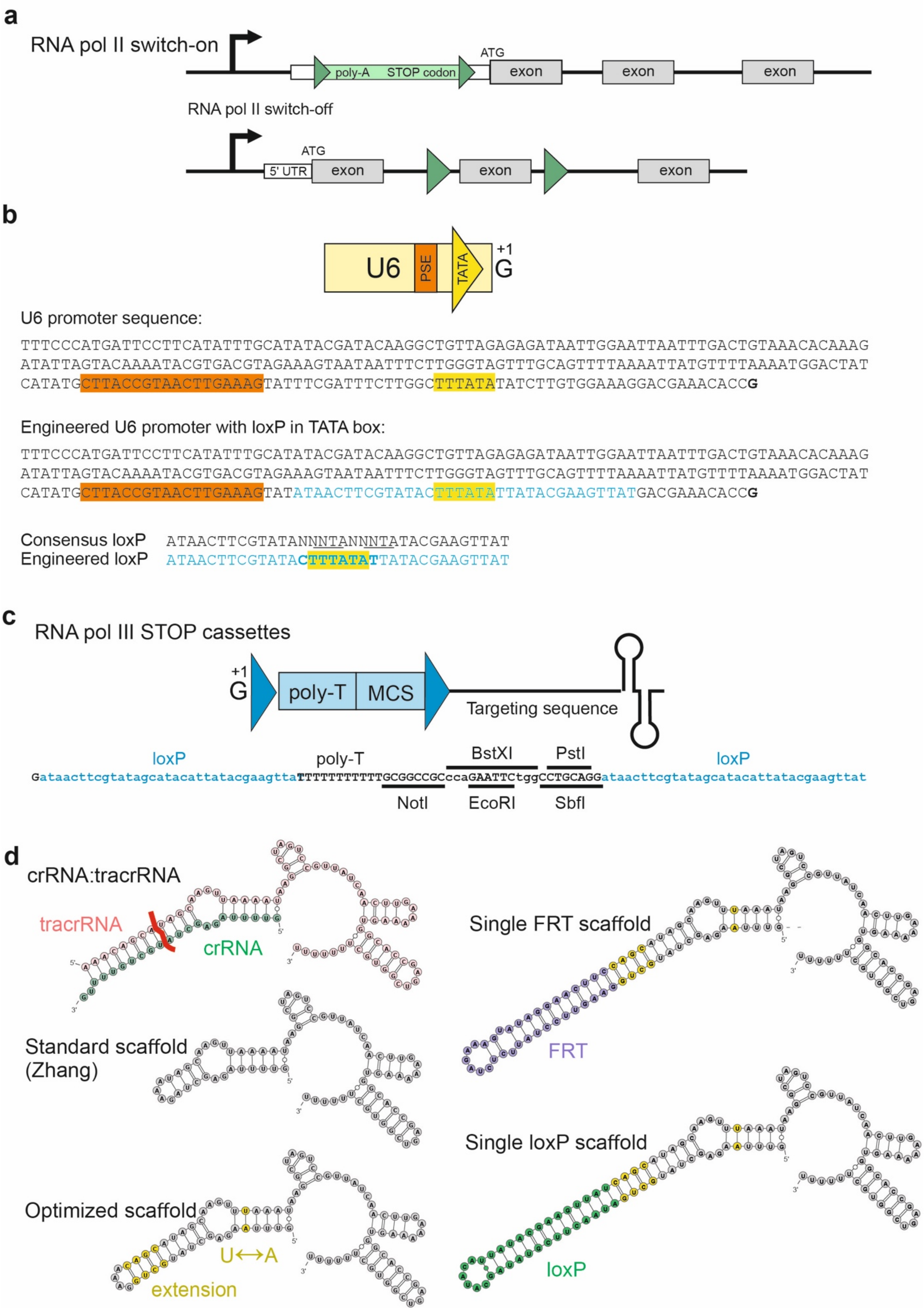

Supplementary Figure 1 – Recombinase-based STOP cassettes. Compare to Figure 1. (**a**) Recombinase-based systems used for RNA polymerase II transcripts. The Switch-on system (top) is based on transcriptional and translational termination signals, typically in a 5'-UTR (white box) flanked by recombinase-recognition sites (triangles). Induction of recombination removes the terminator and allows for expression of downstream sequences. In a switch-off system (bottom) recombinase can be used to remove part of the coding sequence flanked by recombinase-recognition sites (triangles). In both cases recombinase-recognition sites are placed in non-coding regions assuring proper expression in the on-state. (**b**) Modified U6 promoter in an "ON"-state. PSE/orange highlight – proximal sequence element, triangle – modified loxP site containing TATA box, yellow highlight – TATA box sequence, blue – engineered loxP with maintained consensus arm sequence and with variable loop region (NNNTANNN in consensus loxP) replaced with TATA box sequence and surrounding nucleotides (blue bold) as found in U6 promoter. This setup inactivates the promoter if a STOP cassette between two loxP sites disturbs the distance between the TATA-box and proximal sequence element (PSE) essential for transcription. To maintain the distance requirements (17 bp) between TATA and PSE upon cassette excision, the TATA box is inserted within the non-palindromic and less conserved central motif of loxP[22]. (**c**) RNA pol III STOP cassette schematics and corresponding sequence is shown. Triangles and blue sequences represent recombination sites, here loxP sites, however different loxP sequences or FRT sequence can be used. poly-T (polythymidine stretch) – RNA polymerase III transcription termination sequence, MCS – multi-cloning site. Introduction of a STOP cassette into a 5'-loxP location (See Figure 1a), right downstream of the initiating 5' guanosine (G^{+1}), leads to production of a short non-functional transcript consisting of G^{+1}-loxP-poly-U. Upon excision of the STOP cassette, the loxP site is transcribed and followed by a full sgRNA sequence. We hypothesize that the presence of a loxP sequence at the 5'-end of sgRNA either does not interfere with sgRNA function or is degraded *in vivo*[44,45]. (**d**) *In silico* predicted secondary structure of sgRNA scaffolds and of a naturally occurring tracrRNA:crRNA-repeat duplex. Red line indicates the original scaffold proposed by Cong et al.[3].

Supplementary Figure 2

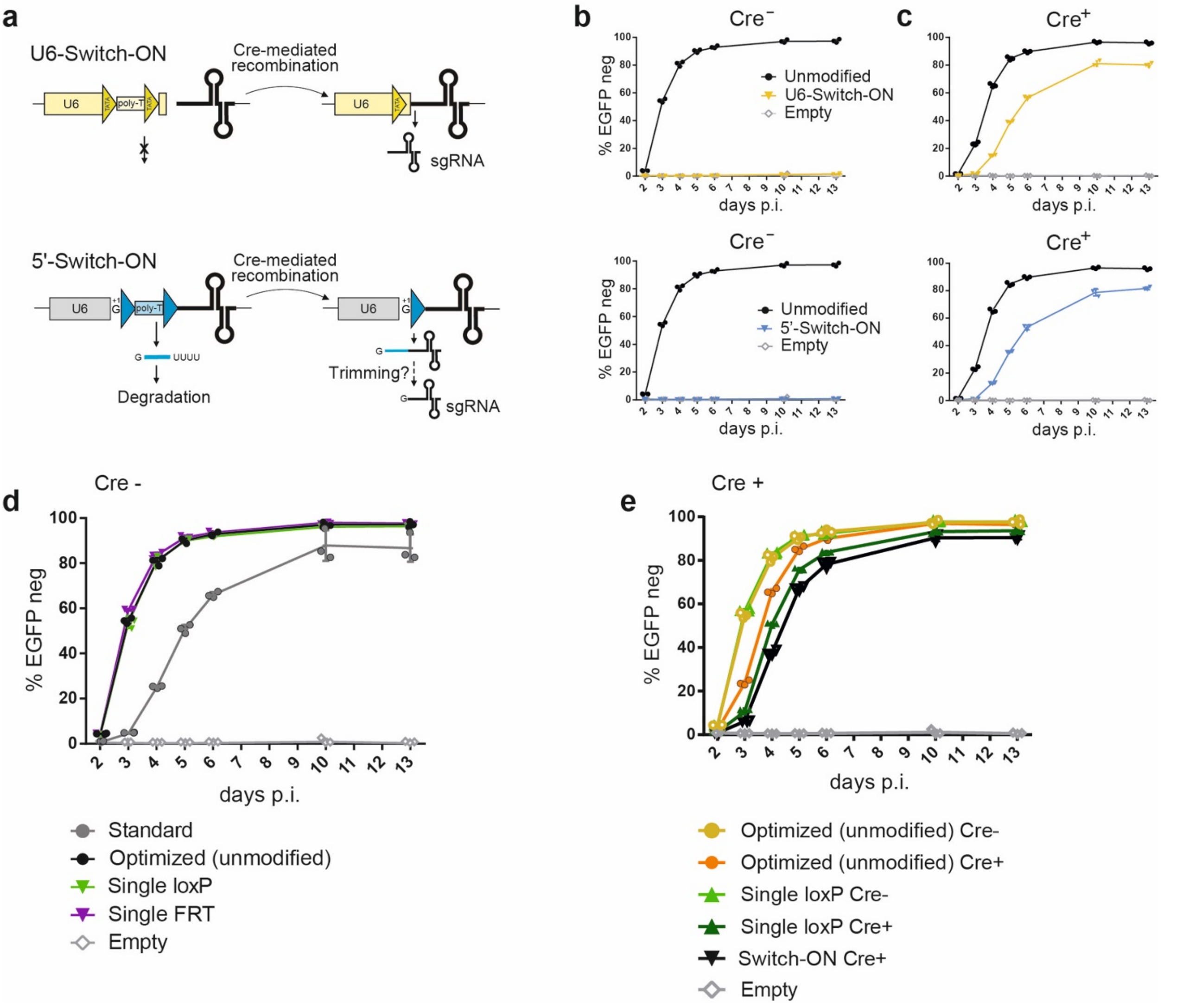

Supplementary Figure 2 – Alternative sgRNA activation systems. See Figures 1 and S1. Activity of switchable sgRNA cassettes with alternative localizations of a STOP cassette within U6 promoter (yellow) and at the 5'-end of sgRNA (blue) is shown. (**a**) Locus architecture of sgRNA induction systems in an "off" (left) and "on" (right) states switchable via recombination. sgRNA and sgRNA-coding sequence is shown as thick line stem-loop structure and recombination sites are represented as triangles. U6 – U6 promoter, poly-T – polythymidine termination signal. G^{+1} – initiating Guanosine required for U6-driven transcription. (**b, c**) Recombinase-inducible sgEGFP1 guide RNAs were introduced into EGFP and Cas9-expressing mES cells without (**b**) or with (**c**) constitutive recombinase expression and EGFP loss was monitored with flow cytometry over 13 days post infection (days p. i.). (**d**) Cre-inducible sgEGFP1 guide RNAs were introduced into EGFP and Cas9-expressing mES cells with inducible Cre-ERT. EGFP loss was monitored using flow cytometry over 13 days p. i.. Cre induction with 4OH-tamoxifen was performed at indicated timepoints. (**b**, **c**, **d**) sgRNA construct with optimized scaffold and no targeting sequence was used as a negative control (empty) and gEGFP1 guide with optimized scaffold as a positive control (unmodified). Error bars represent standard deviation (n = 3). **d**) Comparison of EGFP loss kinetics obtained in EGFP and Cas9-expressing mES cells by sgRNAs with different scaffolds

including recombinase site-containing scaffolds (Fig. 1a single recombination site scaffolds). Two basic sgRNA scaffolds tested are standard sgRNA found in commonly used vectors (e.g. pX330) and the optimized sgRNA scaffold with prolonged repeat:anti-repeat duplex and a T-A base-pair flip preventing premature transcription termination within the scaffold[25]. The optimized scaffold shows higher overall activity and faster kinetics of GFP loss. Most importantly, single loxP and single FRT constructs provided robust deletion efficiency proving that palindromic site-specific recombination targets are tolerated extensions to the sgRNA repeat:antirepeat structure. sgRNA construct with optimized scaffold and no targeting sequence cloned was used as an empty negative control. days p. i. – days post infection. Error bars represent standard deviation (*n* = 3). (**e**) Analysis of activity of various constructs in presence of Cre recombinase; Kinetics of EGFP loss was monitored with flow cytometry over 13 days post infection (days p.i.) with constitutive sgEGFP1 with optimized and single loxP scaffolds and inducible Switch-ON sgEGFP1 in Cre- and Cre+ mES cells expressing Cas9. sgRNA construct with optimized scaffold and no targeting sequence cloned was used as an empty negative control. Note a delay of optimized sgEGFP1 in Cre+ vs. Cre- cell line suggesting lower Cas9 activity in the former. Stronger delay is observed for both single loxP and Switch-ON constructs in Cre+ line while single loxP and optimized have the same kinetics and efficiency in Cre- line. Error bars represent standard deviation (*n* = 3). Source data are provided as a Source Data file.

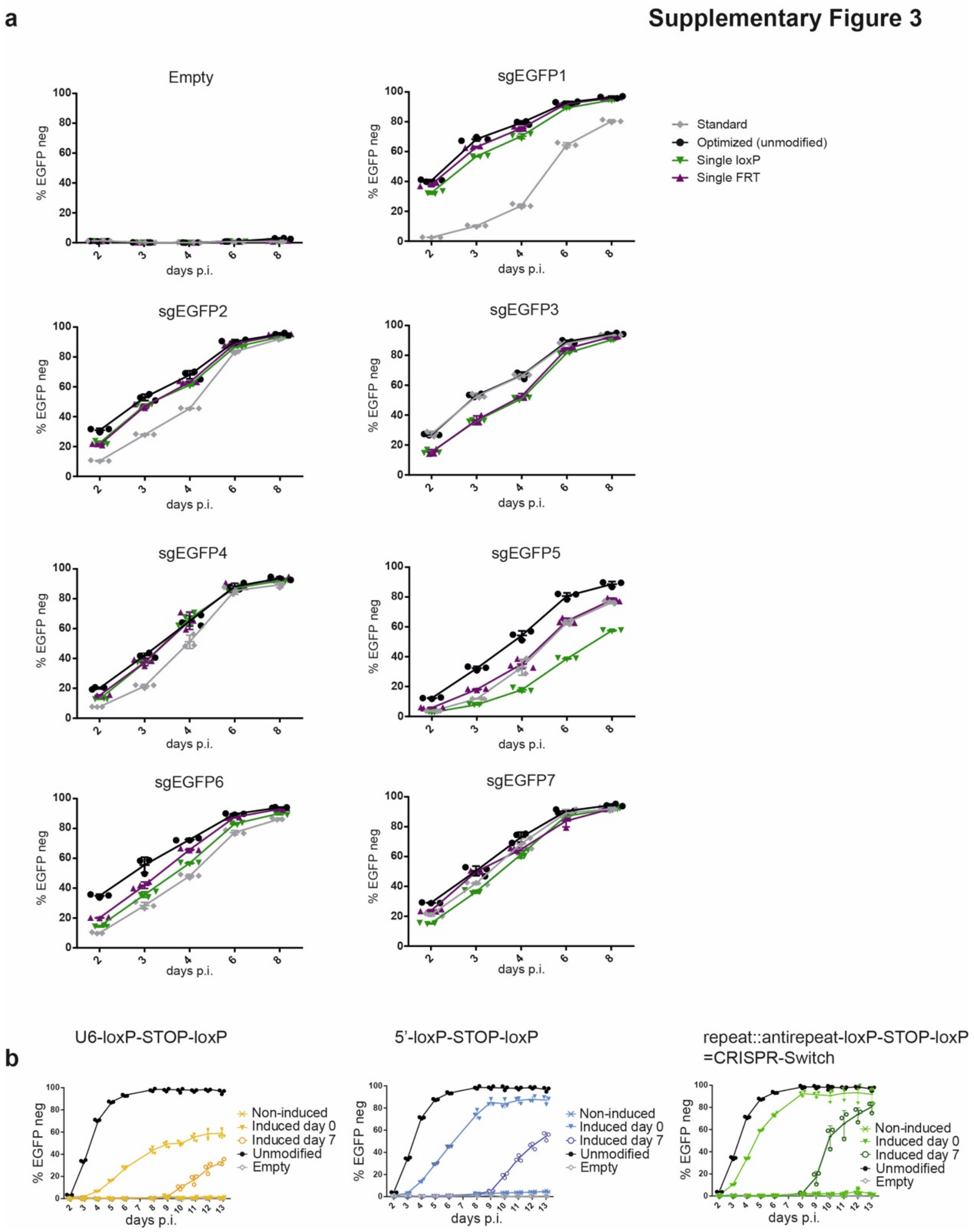

Supplementary Figure 3 – Comparison of EGFP loss kinetics obtained by sgRNAs with different scaffolds. (**a**) EGFP loss was monitored over 8 days post infection with retroviruses encoding sgRNAs against EGFP. Kinetics of EGFP loss was compared for different scaffolds with the same targeting sequence. Percentage of EGFP negative cells was determined using flow cytometry. Error bars represent standard deviation (*n* = 3). (**b**) Comparison of LoxP-STOP-loxP insertions at various locations within the Pol III cassette. Cre-inducible sgEGFP1 guide RNAs were introduced into EGFP and Cas9-expressing mES cells with inducible Cre-ERT2. EGFP loss was monitored using flow cytometry over 13 days p. i.. Cre induction with 4OH-tamoxifen was performed at indicated timepoints. sgRNA construct with optimized scaffold and no targeting sequence was used as a negative control (Empty) and gEGFP1 guide with optimized scaffold as a positive control (Unmodified). Error bars represent standard deviation (*n* = 3). Source data are provided as a Source Data file.

Supplementary Figure 4

a

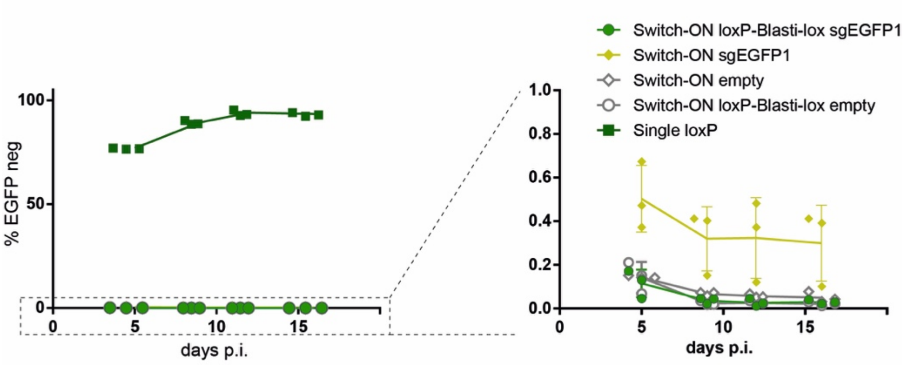

b

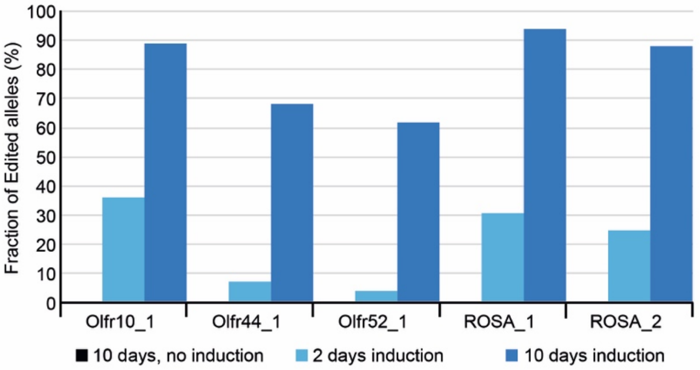

c

Total reads in NGS

	10 days no induction			2 days with induction			10 days with induction		
guide	wildtype	insertions	deletions	wildtype	insertions	deletions	wildtype	insertions	deletions
Olfr10_1	44020	0	0	15456	3590	5169	2365	6848	12295
Olfr44_1	46515	0	0	32654	1010	1565	9253	4552	15151
Olfr52_1	36062	0	0	27125	920	233	5951	4662	4988
ROSA26_1	43976	0	0	25667	0	11356	1929	822	29802
ROSA26_2	50337	0	0	28585	0	9328	3809	314	27991

Supplementary Figure 4 – Further improvement of tightness by antibiotic resistance cassette. (**a**) EGFP loss was measured in Cas9-encoding, Cre-negative mES cells infected with sgEGFP1 guide RNAs with standard Switch-ON cassette and with a modified cassette carrying additionally blasticidin resistance between loxP sites lox-Blasi-lox over 14 days post infection. Empty constructs with no guiding sequence cloned were used as negative controls. CRISPR-Switch cassettes provide tight transcriptional control in the absence of Cre (Figure 1b), however we very low leakiness of the CRISPR-Switch constructs was observed in the inducible Cre-ERT cell line (1.5-4.5 %) measured 13 days post infection, that could have been caused by combined effect of leaky Cre-ERT present in this line and potential spontaneous recombination of viral constructs in bacteria or during packaging. To circumvent this issue, the STOP cassette was modified by introducing a cell culture selectable blasticidin resistance marker between the loxP sites, downstream of polythymidine stretch. Modified cassettes allowed to reduce leakiness to levels present in the empty controls. Error bars represent standard deviation (n = 3). (**b**) Transfection of sgRNAs targeting 5 endogenous loci in mouse ES cells with constitutive expression of Cas9. Next generation sequencing upon maintenance for 10 days in presence and absence of induction as well as induction for 2 days confirms that CRISPR-Switch is fully tight in the OFF-state and highly active in the ON-state. It also illustrates that partial editing obtained in Main Figure 2 was likely due

to incomplete expression of Cas9 and/or CreERT2. (**c**) Raw read table of the experiment shown in (b). Source data are provided as a Source Data file.

Supplementary Figure 5

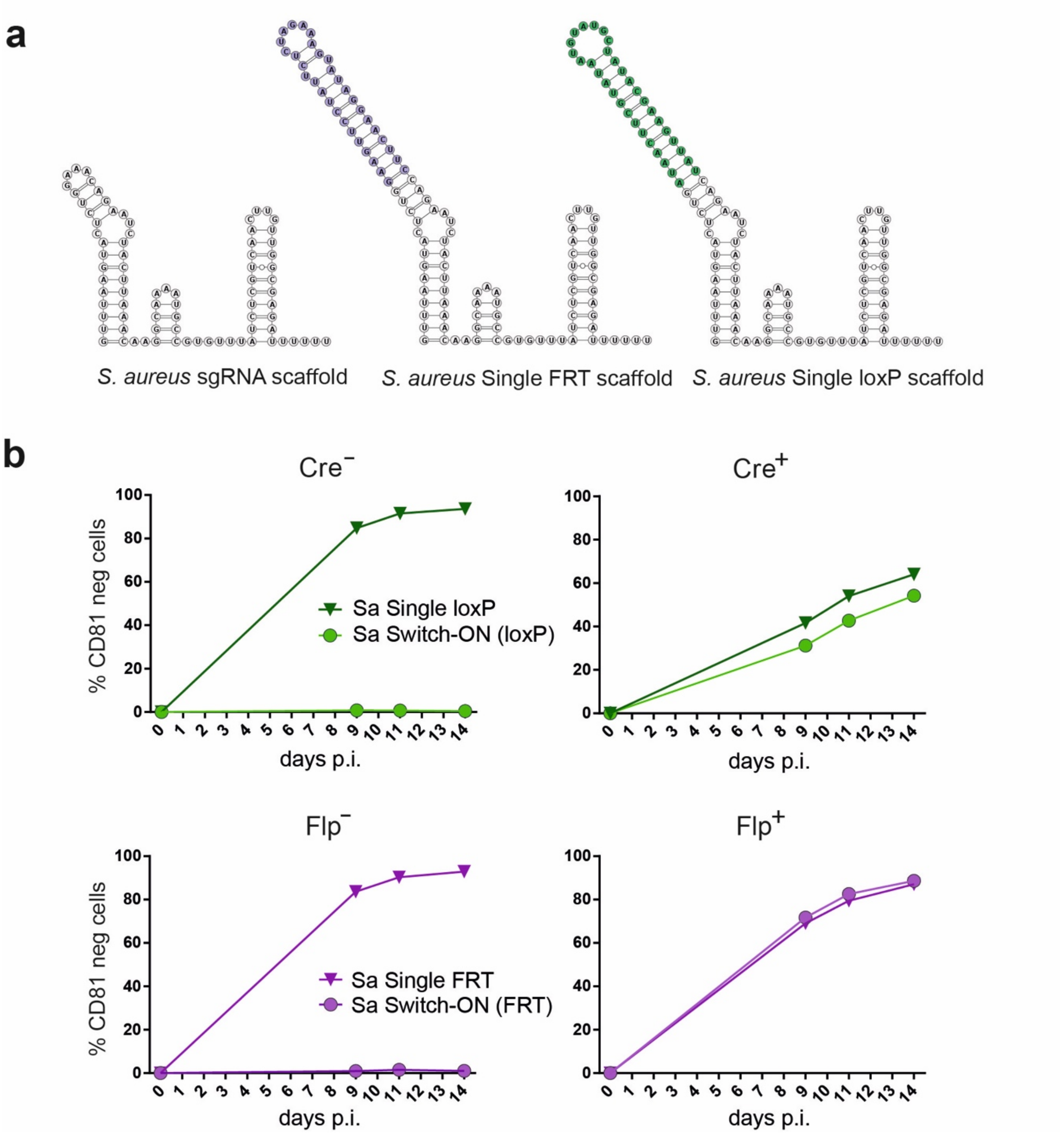

Supplementary Figure 5 – Recombinase-based inducible sgRNAs for *Staphylococcus aureus* CRISPR/Cas9. (**a**) *In silico* modelling of the predicted secondary structure of the unmodified *S. aureus* (Sa) sgRNA scaffold and the single FRT and single loxP scaffolds are shown. A single FRT or loxP sequence remains in the repeat:anti-repeat stem-loop after recombination. (**b**) WT (left; Cre-, Flp-) and Cre recombinase- or Flippase-expressing (right; Cre+, Flp+) A375 human melanoma lines were infected with a lentivirus encoding Sa Cas9 and modified Sa sgRNAs targeting CD81, in which the sgRNA scaffold either contained a transcription STOP cassette flanked by loxP/FRT sites (Sa Switch-ON or Sa Switch-ON (FRT)) or a single loxP/FRT site (Sa single loxP or Sa single FRT; compare with Figure 1). CD81 protein loss was measured with flow cytometry using APC-coupled anti-CD81 antibody over 14 days post infection (p. i.).

Supplementary Figure 6

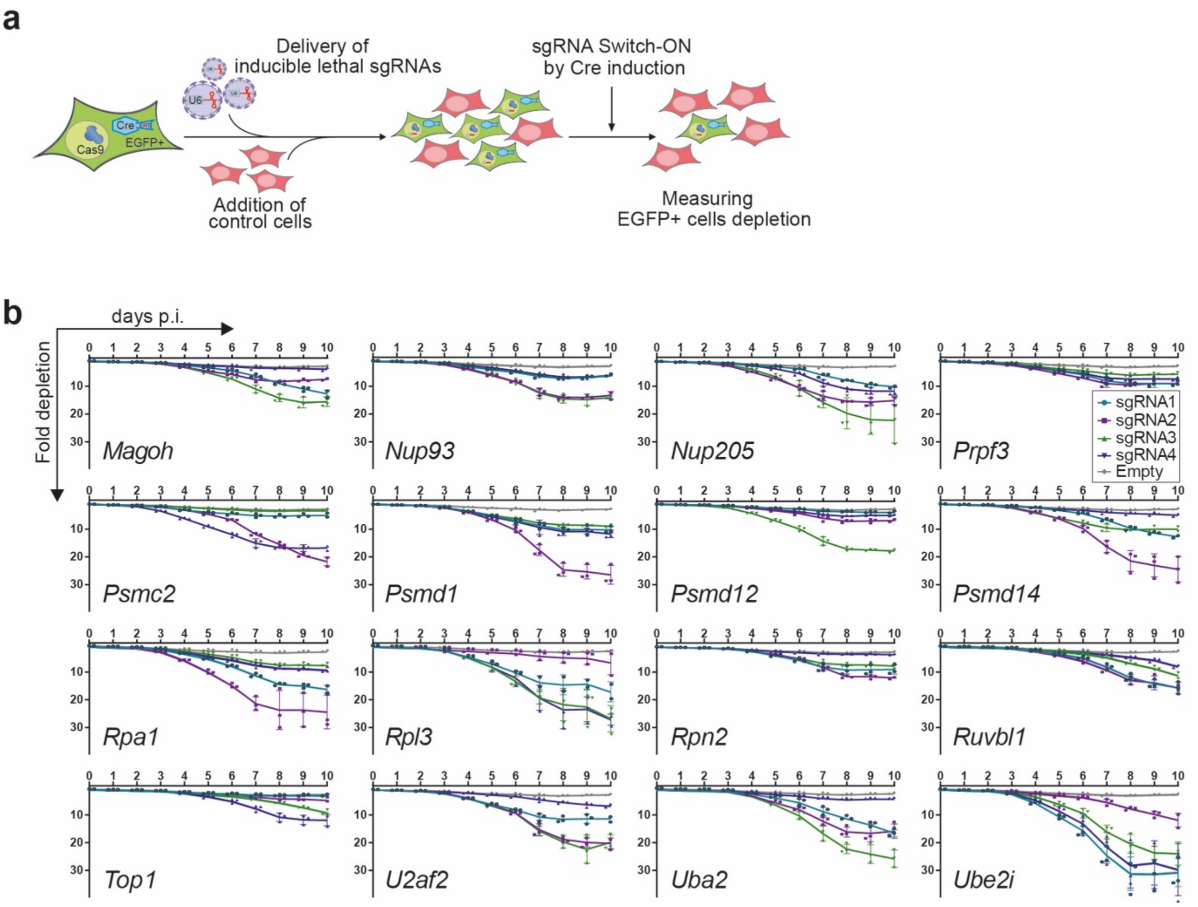

Supplementary Figure 6 – targeting essential genes using CRISPR-Switch-ON (**a**) Experimental setup. EGFP-positive, Cas9 and Cre-ERT-encoding mES cells were infected with Cre-inducible sgRNA against essential genes and mixed with mCherry-positive Cas9 and Cre-ERT-encoding mES control cells. After induction of sgRNAs, depletion of EGFP-positive cells was monitored with flow cytometry. (**b**) Depletion of EGFP-positive cells caused by editing of essential genes was monitored for 10 days upon induction. Depletion was calculated as a fold change in EGFP-positive over mCherry positive cell count ratio between day of infection (day 0) and day of measurement. EGFP-positive cells infected with empty sgRNA vector were used as negative control. Error bars represent standard deviation (*n* = 3). Source data are provided as a Source Data file.

Supplementary Figure 7

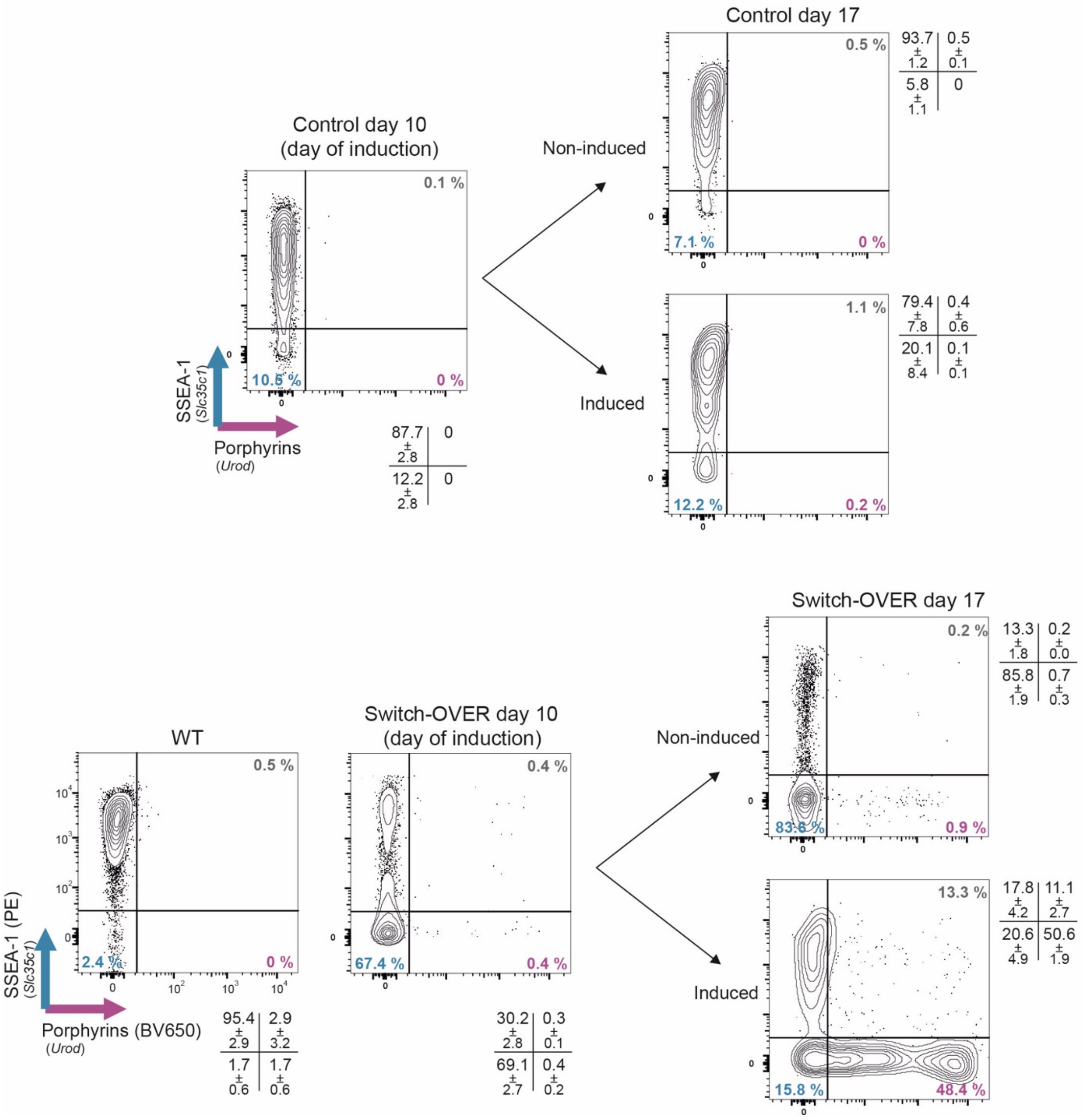

Supplementary Figure 7 – Recombinase-mediated consecutive editing *in vitro* I. See Figure 4b. Control sample was infected with a construct encoding two non-targeting sgRNAs (top). Consecutive deletion of *Slc35c1* and *Urod* in mES cells is shown in the bottom panel. SSEA-1 loss and porphyrin accumulation caused by Urod mutational effect was monitored by flow cytometry over 17 days. At day 10 post infection the cells were divided into two wells. In one culture Cre-ERT was induced leading to inactivation of sgRNA against *Slc35c1* and activation of sgRNA against *Urod*. Results for both cultures (induced and non-induced) are shown. Plots show representative samples of a triplicate (*n* = 3). Note that WT and control samples show small populations of Ssea-1 negative cells. Source data are provided as a Source Data file.

Supplementary Figure 8

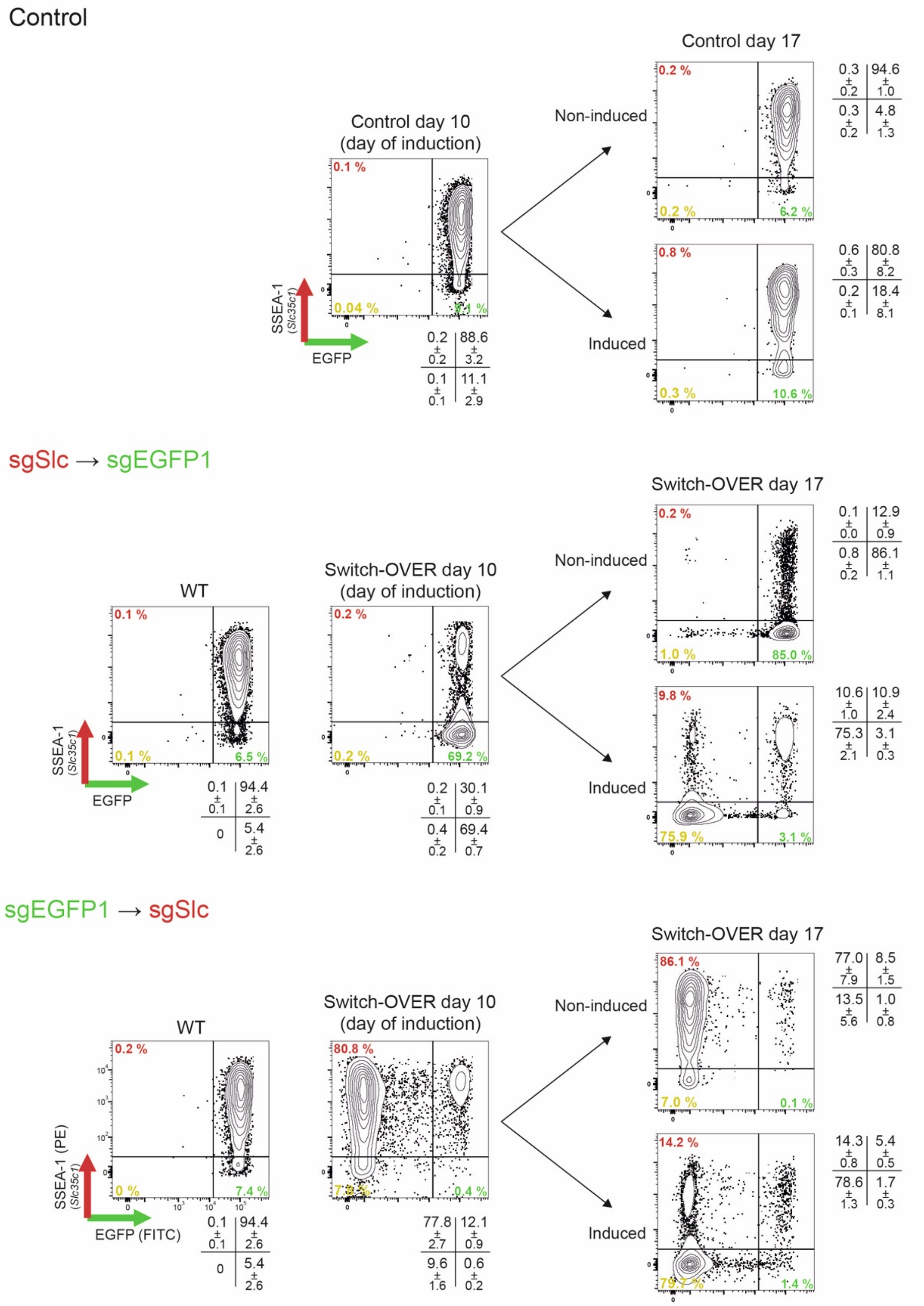

Supplementary Figure 8 – Recombinase-mediated consecutive editing *in vitro* II. See Figures 3b and S7. Control sample was infected with a construct encoding two non-targeting sgRNAs (top). Consecutive deletion of *Slc35c1* and EGFP in mES cells is shown in the lower panels. Two CRISPR-Switch OVER constructs with alternative order of sgRNA expression were tested. SSEA-1 and EGFP loss was monitored by flow cytometry over 17 days. At day 10 post infection the cells were divided into two wells. In one culture Cre-ERT was induced leading to inactivation of a first sgRNA and activation of a second sgRNA. Results for both cultures (induced and non-induced) are shown. Plots show representative samples of a triplicate (*n* = 3). Note that WT and control samples show small populations of SSEA-1 negative cells. Source data are provided as a Source Data file.

Supplementary Figure 9

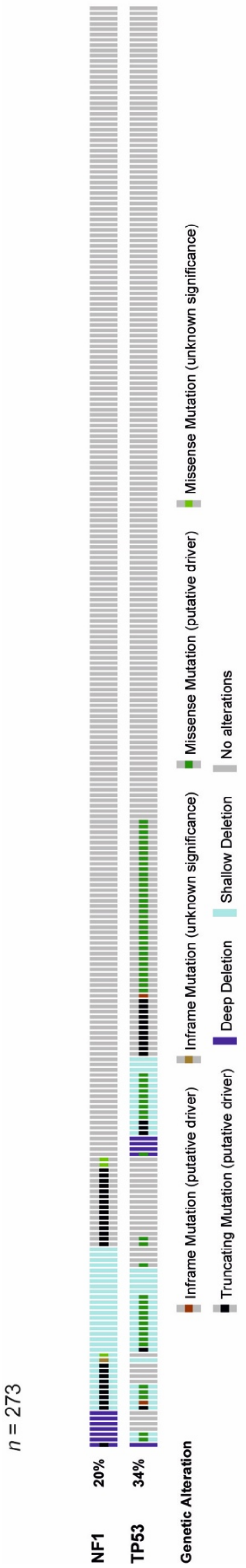

Supplementary Figure 9 – TP53 and NF1 mutations in GBM. Oncoprint (http://www.cbioportal.org/oncoprinter) shows occurrence and type of mutations found in TP53 and NF1 in glioblastoma multiforme patients.

Supplementary Figure 10

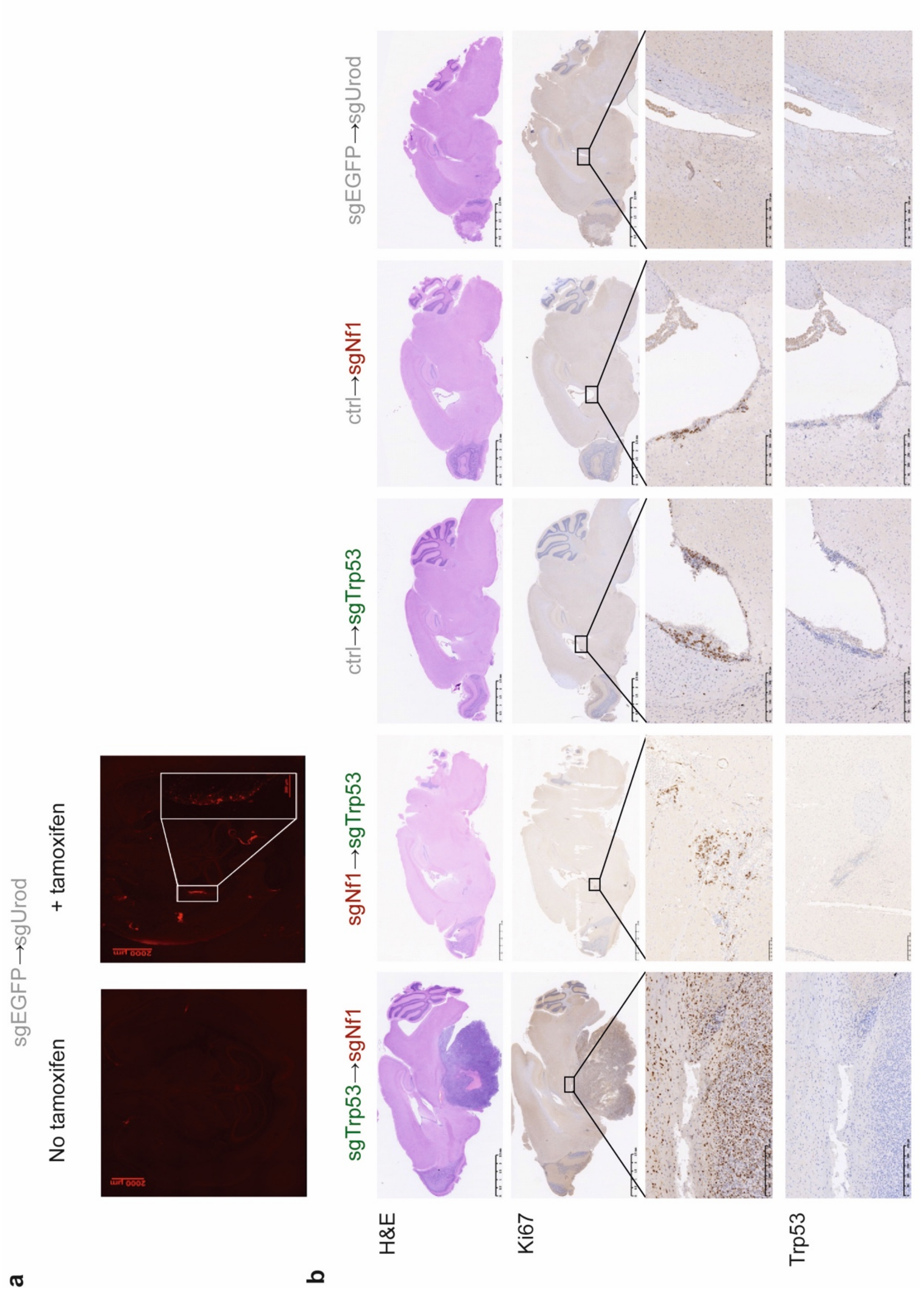

Supplementary Figure 10 – Recombinase-mediated consecutive editing *in vivo.* See Figure 4c, d. Representative images of Cas9-GFP; Nestin-CreERT2 mouse brains transduced with indicated dual sgRNA lentivirus. Mice were transduced into the right ventricle at postnatal day p0 and sgRNA recombination was induced at P21 using tamoxifen. (**a**) Fluorescence microscopy images of sections from brains transduced with sgEGFP-to-sgUrod constructs with and without induction of recombination. (**b**) Brain section were stained with H&E, anti-Ki67 and anti-P53 antibodies showing examples of macroscopic, microscopic tumors as well as hyperproliferation.

Supplementary Figure 11

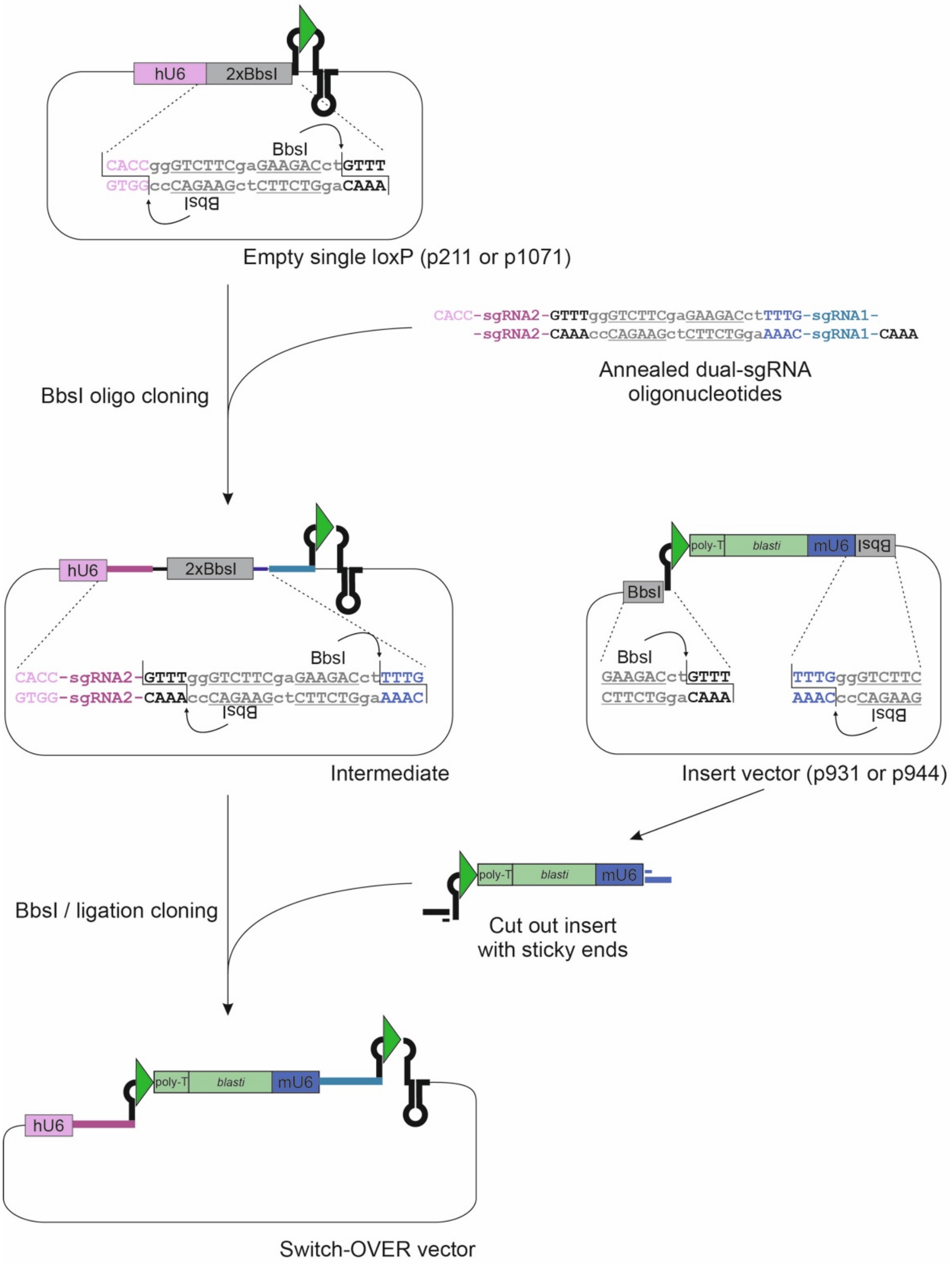

Supplementary Figure 11 – Construction of Switch-OVER vectors. Two-step cloning strategy used for construction of consecutive editing vectors is shown. In a first step, single loxP construct is opened with BbsI (BsmBI for lentiviral vectors) producing sticky ends as indicated. Annealed DNA oligonucleotides with compatible sticky ends are cloned into single loxP backbone as in regular procedure of sgRNA oligo cloning (see Methods section on molecular cloning). Between the sticky ends (pink and black) oligos encode: 20 nt sgRNA2 targeting sequence (magenta), 1st adaptor for further cloning step (black: sticky end compatible with sgRNA scaffold), dual BbsI restriction cassette (gray, BsmBI for lentiviral vectors), 2nd adaptor for further cloning step (dark blue: sticky compatible with mU6), 20 nt sgRNA1 targeting sequence (cyan). Resulting intermediate construct is opened with BbsI (BsmBI for lentiviral vectors) producing sticky ends as indicated. BbsI-cut-out insert (from an insert vector containing a part of sgRNA single loxP scaffold, poly-T STOP signal, optional antibiotic resistance cassette (*blasti*) and mouse U6 promoter (mU6)) is cloned into the open intermediate vector using regular restriction/ligation protocol resulting in a ready Switch-OVER construct.

Supplementary Table 1 – Basic plasmid constructs

ID	Name	Description	Construction notes
**p21**	sgRNA standard	Retroviral vector for sgRNA expression: U6-BbsIx2- standard scaffold[3] (pX330); neoR	
**p76**	sgRNA optimized (unmodified control)	Retroviral vector for sgRNA expression: U6-BbsIx2- optimized scaffold[22]; neoR	
**p204**	U6-Switch-ON	Retroviral Switch-ON vector; neoR	PCR mutagenesis / ligation with CR231/CR229 on p76
**p205**	5'-Switch-ON	Retroviral Switch-ON vector; neoR	oligo cloning: p76 + CR297/Phos-CR298/CR311/Phos-CR312
**p206**	Switch-ON	Retroviral Switch-ON vector; neoR	PCR mutagenesis / ligation with CR237/CR235 on p76
**p207**	Switch-ON (FRT)	Retroviral Switch-ON vector; neoR	PCR mutagenesis / ligation with CR240/CR238 on p76
**p208**	Single FRT	Retroviral vector; neoR	PCR mutagenesis / ligation with CR243/CR244 on p76
**p211**	Single loxP	Retroviral vector; neoR	PCR mutagenesis / ligation with CR316/CR317 on p76
**p213**	Switch-OFF	Retroviral Switch-OFF vector; neoR	PCR mutagenesis / ligation with CR314/CR315 on p211
**p223**	Pulse-Switch	Lentiviral Pulse-Switch vector; blastiR	Gibson: PCR CR587/CR588 (EF1a-cas9-p2a-blasti) on p468 and PCR CR589/CR590 (backbone-U6-scaffold) on p468
**p468**	Lenti-sgRNA+Cas9	Lentiviral vector for Cas9 and sgRNA with optimized scaffold expression: U6-2xBsmBI-optimized scaffold-EF1a-Cas9-p2A-blasti (Julian Jude)	
**p620**	Switch-ON lox-Blasti-lox	Retroviral Switch-ON vector with blastiR within STOP cassette; blastiR, neoR	Gibson: PCR CR1551/CR1552 on p206 + PCR CR1555/CR1556 on p223; mutagenesis to remove BpiI site from blasti by PCR CR1571/CR1572
**p869**	Switch-OVER insert: PGK-Blasti	insert vector for Switch-OVER: Single loxP-STOP-PGK-Blasti-mU6	Gibson: PCR CR2108/CR2109 on pUC19, PCR CR2110/CR2111 on p620 and PCR CR2112/CR2113 on Addgene 53187
**p931**	Switch-OVER insert: EF1a-Blasti	insert vector for Switch-OVER: Single loxP-STOP-EF1a-Blasti-mU6	Gibson: PCR CR2162/CR2163 on p869 + PCR CR2164/CR2165 on p223
**p944**	Switch-OVER insert: EF1a-Puro	insert vector for Switch-OVER: Single loxP-STOP-EF1a-Puro-mU6	Gibson: PCR CR2183/CR2184 on p931 and PCR CR2185/CR2186 on Addgene 62988
**p1071**	Lenti-Single loxP	Lentiviral vector - entry vector for Lentiviral Switch-OVER	PCR mutagenesis / ligation with CR2433/CR2434 on p223
**XPR_206**	All-in-one SaCas9 + sgRNA	Lentiviral vector with S. aureus Cas9 and sgRNA Addgene #96920	
**RDA_068**	SaCas9 Single loxP	Retroviral vector; S. aureus	RD_1 into XPR_206 via KpnI and EcoRI restriction/ligation
**RDA_070**	SaCas9 Single FRT	Retroviral vector; S. aureus	RD_2 into XPR_206 via KpnI and EcoRI restriction/ligation
**RDA_098**	SaCas9 Switch-ON	Retroviral Switch-ON vector; S. aureus	RD_3 into XPR_206 via KpnI and EcoRI restriction/ligation
**RDA_100**	SaCas9 Switch-ON (FRT)	Retroviral Switch-ON vector; S. aureus	RD_4 into XPR_206 via KpnI and EcoRI restriction/ligation

Supplementary Table 2 – Synthetic DNA sequences used for backbone construction

ID	SEQUENCE
CR229	CGCAAAAAAAAAAAAATAACTTCGTATAATATAAAGTATACGAAGTTATATACTTTCAAGTTACGGTAAGCATATGATAGTC
CR231	GCCGCCCAGAATTCTGGCCTGCAGGATAACTTCGTATACTTTATATTATACGAAGTTATGACGAAACACCGGGTCTTCG
CR235	CGCAAAAAAAAAAAAATAACTTCGTATAATGTATGCTATACGAAGTTATCAGCATAGCTCTTAAACAGGTCTTCTCT
CR237	GCCGCCCAGAATTCTGGCCTGCAGGATAACTTCGTATAGCATACATTATACGAAGTTATCAGCATAGCAAGTTTAAATAAGGCTAGTCC
CR238	CGCAAAAAAAAAAAGAAGTTCCTATACTTTCTAGAGAATAGGAACTTCCAGCATAGCTCTTAAACAGGTCTTCTCT
CR240	GCCGCCCAGAATTCTGGCCTGCAGGGAAGTTCCTATTCTCTAGAAAGTATAGGAACTTCCAGCATAGCAAGTTTAAATAAGGCTAGTCC
CR243	CTTCCAGCATAGCAAGTTTAAATAAGGCTAGTCCG
CR244	TTCCTATACTTTCTAGAGAATAGGAACTTCCAGCATAGCTCTTAAACAGGTCTTCTCT
CR297	CACCGATAACTTCGTATAGCATACATTATACGAAGTTATTTTTTTTTTTTGCGGCCGCCCAGAATTCTGGCCT
CR298	GCAGGATAACTTCGTATAGCATACATTATACGAAGTTATGGGTCTTCGCTCGAGAGAAGACCT
CR311	GGCGGCCGCAAAAAAAAAAAAATAACTTCGTATAATGTATGCTATACGAAGTTATC
CR312	AAACAGGTCTTCTCTCGAGCGAAGACCCATAACTTCGTATAATGTATGCTATACGAAGTTATCCTGCAGGCCAGAATTCTG
CR314	TAATGTATGCTATACGAAGTTATCTGCAGAATTGGCGCACGC
CR315	TACGAAGTTATACAAATGGCTCTAGAGGTACCCG
CR316	TACGAAGTTATCAGCATAGCAAGTTTAAATAAGGCTAGTCCG
CR317	TAATGTATGCTATACGAAGTTATCAGCATAGCTCTTAAACAGGTCTTCTCT
CR587	TTCGTATAGCATACATTATACGAAGTTATTTTTTTTTTTTTTAGCTAGGTCTTGAAAGGAGTGGG
CR588	AAGTTGATAACGGACTAGCCTTATTTAAACTTGCTATGCTGATAACTTCGTATAATGTATGCTATACGAAGTTATCCGATAAGCTTGATATCGAATTCTTAGCCC
CR589	ATAAGGCTAGTCCGTTATCAACTTGAAAAAGTGGCACCGAGTCGGTGCTTTTTTGCTAATCAACCTCTGGATTACAAAATTTGTGAAAGA
CR590	CTTCGTATAATGTATGCTATACGAAGTTATCAGCATAGCTCTTAAACAGAGACGTTAC
CR1551	CATATTGGCTGCAGGTCGAAAGG
CR1552	TGAGGGGATCCGTCGACC
CR1555	GCCTTTCGACCTGCAGCCAATATGGCCAAGCCTTTGTCTCAAGAAGA
CR1556	TGCAGGTCGACGGATCCCCTCAGCCCTCCCACACATAACCAGA
CR1571	CCATCTCTGAGGATTACAGCGTCGCCAGCGCAGCTCTCTCTAG
CR1572	GACGCTGTAATCCTCAGAGATGGGGATGCTGTTGATTGTAGCCGTTG
CR2183	TTGTACTCGGTCATGGTGGCAGCGCTCTAG
CR2184	AAGCCCGGTGCCTGAGATCCGACGCCGCC
CR2185	AGCGCTGCCACCATGACCGAGTACAAGCCCACG
CR2186	CGGCGTCGGATCTCAGGCACCGGGCTTG
CR2433	ACATTATACGAAGTTATCAGCATAGCAAGTTTAAATAAGGCTAG
CR2434	ATGCTATACGAAGTTATCAGCATAGCTCTTAAACAGAGACGTTAC
CR2108	TTTGGGGTCTTCGACTCTAGAGGATCCCCGGGT
CR2109	AAACAGGTCTTCGACCTGCAGGCATGCAAGC

CR2110	ATGCCTGCAGGTCGAAGACCTGTTTAAGAGCTATGCTGATAACT
CR2111	CGGCGTCGGATCTCAGCCCTCCCACACATAACC
CR2112	TGGGAGGGCTGAGATCCGACGCCGCCATCT
CR2113	TCCTCTAGAGTCGAAGACCCCAAACAAGGCTTTTCTCCAAGGG
CR2162	GAGCGCTGCCACCATGGCCAAGCCTTTGTCTCAAG
CR2163	AATTCCCACTCCACGCGTCAATTCTGGGCG
CR2164	GAATTGACGCGTGGAGTGGGAATTGGCTCCG
CR2165	AGGCTTGGCCATGGTGGCAGCGCTCTAGAACC
**RD_1**	GGTACCGAGGGCCTATTTCCCATGATTCCTTCATATTTGCATATACGATACAAGGCTGTTAGAGAGATAATTAGAATTAATT<br>TGACTGTAAACACAAAGATATTAGTACAAAATACGTGACGTAGAAAGTAATAATTTCTTGGGTAGTTTGCAGTTTTAAAATT<br>ATGTTTTAAAATGGACTATCATATGCTTACCGTAACTTGAAAGTATTTCGATTTCTTGGCTTTATATATCTTGTGGAAAGGA<br>CGAAACACCGAGGCTGTGGTGAAGACCTTCCGTTTAAGTACTCTGATAACTTCGTATAATGTATGCTATACGAAGTTATCAG<br>AATCTACTTAAACAAGGCAAAATGCCGTGTTTATCTCGTCAACTTGTTGGCGAGATTTTTTGAATTC
**RD_2**	GGTACCGAGGGCCTATTTCCCATGATTCCTTCATATTTGCATATACGATACAAGGCTGTTAGAGAGATAATTAGAATTAATT<br>TGACTGTAAACACAAAGATATTAGTACAAAATACGTGACGTAGAAAGTAATAATTTCTTGGGTAGTTTGCAGTTTTAAAATT<br>ATGTTTTAAAATGGACTATCATATGCTTACCGTAACTTGAAAGTATTTCGATTTCTTGGCTTTATATATCTTGTGGAAAGGA<br>CGAAACACCGAGGCTGTGGTGAAGACCTTCCGTTTAAGTACTCTGGAAGTTCCTATTCTCTAGAAAGTATAGGAACTTCCAG<br>AATCTACTTAAACAAGGCAAAATGCCGTGTTTATCTCGTCAACTTGTTGGCGAGATTTTTTGAATTC
**RD_3**	GGTACCGAGGGCCTATTTCCCATGATTCCTTCATATTTGCATATACGATACAAGGCTGTTAGAGAGATAATTAGAATTAATT<br>TGACTGTAAACACAAAGATATTAGTACAAAATACGTGACGTAGAAAGTAATAATTTCTTGGGTAGTTTGCAGTTTTAAAATT<br>ATGTTTTAAAATGGACTATCATATGCTTACCGTAACTTGAAAGTATTTCGATTTCTTGGCTTTATATATCTTGTGGAAAGGA<br>CGAAACACCGAGGCTGTGGTGAAGACCTTCCGTTTAAGTACTCTGATAACTTCGTATAATGTATGCTATACGAAGTTATTTT<br>TTTCGGTAAGGGGAGTAAACCGCGCGAATTACAGTTGCGACGTCACGATCAAATCTGGCAGATCGGGTTTACAGGCCAGGAC<br>TGGACAGGTCATTTGCGGTCCACGTGACGACTACTCAGCGTCAGTTATGAGAGAGCATGTTCCAAAACGGGAGCATAGCAGA<br>GGCAAGCACAATGGTCCTCGTGTCTGGGGGTCGCCTGAGTGGGACCATTTTGGATCGAGCGTAGTAATGAGGTTCCCACGTG<br>AGCAGCACGCGATGACGGGTCAACCCACGAATGAACGGTGGACAGTACGGTGAACTCTTTTTTATAACTTCGTATAATGTAT<br>GCTATACGAAGTTATCAGAATCTACTTAAACAAGGCAAAATGCCGTGTTTATCTCGTCAACTTGTTGGCGAGATTTTTTGAA<br>TTC
**RD_4**	GGTACCGAGGGCCTATTTCCCATGATTCCTTCATATTTGCATATACGATACAAGGCTGTTAGAGAGATAATTAGAATTAATT<br>TGACTGTAAACACAAAGATATTAGTACAAAATACGTGACGTAGAAAGTAATAATTTCTTGGGTAGTTTGCAGTTTTAAAATT<br>ATGTTTTAAAATGGACTATCATATGCTTACCGTAACTTGAAAGTATTTCGATTTCTTGGCTTTATATATCTTGTGGAAAGGA<br>CGAAACACCGAGGCTGTGGTGAAGACCTTCCGTTTAAGTACTCTGGAAGTTCCTATTCTCTAGAAAGTATAGGAACTTCTTT<br>TTTCGGTAAGGGGAGTAAACCGCGCGAATTACAGTTGCGACGTCACGATCAAATCTGGCAGATCGGGTTTACAGGCCAGGAC<br>TGGACAGGTCATTTGCGGTCCACGTGACGACTACTCAGCGTCAGTTATGAGAGAGCATGTTCCAAAACGGGAGCATAGCAGA<br>GGCAAGCACAATGGTCCTCGTGTCTGGGGGTCGCCTGAGTGGGACCATTTTGGATCGAGCGTAGTAATGAGGTTCCCACGTG<br>AGCAGCACGCGATGACGGGTCAACCCACGAATGAACGGTGGACAGTACGGTGAACTCTTTTTTGAAGTTCCTATTCTCTAGA<br>AAGTATAGGAACTTCCAGAATCTACTTAAACAAGGCAAAATGCCGTGTTTATCTCGTCAACTTGTTGGCGAGATTTTTTGAA<br>TTC

Supplementary Table 3 – sgRNA targeting sequences

EGFP	sgEGFP1	GAGCTGGACGGCGACGTAAA
	sgEGFP2	CCCATCCTGGTCGAGCTGGA
	sgEGFP3	GCCGTCCAGCTCGACCAGGA
	sgEGFP4	CTTCAGGGTCAGCTTGCCGT
	sgEGFP5	GGTGGTCACGAGGGTGGGCC
	sgEGFP6	GGAGCGCACCATCTTCTTCA
	sgEGFP7	GAAGGGCATCGACTTCAAGG
Magoh	sgRNA1	GAGTTTGAATTCCGACCTGA
	sgRNA2	GGACTGGTTGACATCAATAA
	sgRNA3	GCCGGCCCACGCGATCAGGA
	sgRNA4	TTGTGTTTTAGGGATCCGGA
Nf1	sgRNA1	TTGTCTCGCCGTATGAAGCA
	sgRNA2	TGCTTCATACGGCGAGACAA
Nup93	sgRNA1	GCATCTCTAATGGATACTGA
	sgRNA2	GTGTCCTTCACAGGCTCGAG
	sgRNA3	TGTCTGCTATTGAAGAGTCC
	sgRNA4	GCTTGTGATAGAAAGTCGAG
Nup205	sgRNA1	TTTTATACTGGGATGGAAAG
	sgRNA2	GCAGCTTGTTGACACTGCGA
	sgRNA3	CATTTCCCTGGCCTCACCAG
	sgRNA4	CTTTATAGACCAAAGCACAG
Prpf3	sgRNA1	TCTTCTACCTCCTCAAAACG
	sgRNA2	GGAGCTTTGTTAGCATGCCA
	sgRNA3	GGCTGAATAGTGTTGCCGAT
	sgRNA4	GATCTCAAAGGAATCATCGG
Psmc2	sgRNA1	GCTGTGTAATCACCGAGACG
	sgRNA2	CTTGGAATCGAGCCTCCAAA
	sgRNA3	CTTCCGGTGGGGAAGAGAAA
	sgRNA4	GGACCAGCGGAAAACCAAAG
Psmd1	sgRNA1	GAGACTCCTCAAAAGCCCCC
	sgRNA2	AAACACTTTCGAGGCCACCA
	sgRNA3	GGCAGTAATAGACAACACAG
	sgRNA4	GATCCTGGAGAAGTGTCCTT
Psmd12	sgRNA1	TTATGCTTCTGTCAAAACGA
	sgRNA2	GGAAGTGGACTACAGCGCCA
	sgRNA3	GCAGTGCTGTACTTACGTGG
	sgRNA4	GCGCATTGTGAAGATGGAAG
Psmd14	sgRNA1	CCTTACCTTTAGCAAGGCCA
	sgRNA2	ATTAGACCCATAACTTCCAT
	sgRNA3	ACCTACAGATGCTCCTGCCG
	sgRNA4	TTAGGATCCCAAACGTCATT
Rpa1	sgRNA1	TATTCCCTGTAGAAATGGGA
	sgRNA2	GCACCTGGAGTAATTCCCGG
	sgRNA3	GTTCCGGCAGGTTTTCCAAA
	sgRNA4	CTACAGGGAATAGGTCACCC
Rpl3	sgRNA1	CCGAACAGAGATTAACAAGA
	sgRNA2	TTTTGGCCCATAGTCCTTGC
	sgRNA3	GCGAGTACTTACTCTTCGTA
	sgRNA4	CCGCATCATTGCCCACACTC
Rpn2	sgRNA1	CATCTGGCACCTGTACCCCA
	sgRNA2	GCTCGGCTGGATGAACTAGG
	sgRNA3	AGGGCTTCATTGGATGCCAA
	sgRNA4	CTACAAGCTCATGGACCACG
Ruvbl1	sgRNA1	AACACCTTGCCAGTTCCAGG
	sgRNA2	ACATCTAGATAAACTTCGAG
	sgRNA3	CCACTTGGCTCACCTCAGAG
	sgRNA4	CCACCTCTCTCGCGTTCTCC
Slc35c1	sgSlc35c1	GAGAAGAACGTTGAACACGG
Top1	sgRNA1	TTTCTTTCCTCAGATCGAAG
	sgRNA2	GGTTCATCTTTAATTCGTGG
	sgRNA3	GGGCACACTCACCATTCAAT
	sgRNA4	CACGCCGGCCGACATGAGCG
Trp53	sgRNA1	GAAGTCACAGCACATGACGG
	sgRNA2	ACAAAATTACAGACCTCGGG
U2af2	sgRNA1	TCGTCGTCTCCTATCCCGAG
	sgRNA2	TCTGACCATAGGCCATTGCG
	sgRNA3	GTACCTCAGTAATGCCAAAA
	sgRNA4	CAGCTCAACGAGAATAAACA
Uba2	sgRNA1	CCAGACACATCCTATTCACG
	sgRNA2	GTCACCCTAAGCCTACCCAG
	sgRNA3	GCAGTCCCGCTCTCAATGAG
	sgRNA4	GCTCGAGCATCTAATGAAGA
Ube2i	sgRNA1	GAGTGCTCCTTACAAAAGGG
	sgRNA2	GCTACTCCATACCTGTTTGA
	sgRNA3	CGCTATCCCTGGAAAGAAGG
	sgRNA4	TCCCTCCCCACAGACTCCAT
Urod	sgUrod	GGGCGGCAGTTCAAGCACCA
ctrl	sgRNA1	GTAGCGAACGTGTCCGGCGT
	sgRNA2	GACCGGAACGATCTCGCGTA

Supplementary Table 4 - Mouse ON target forward oligos for sgRNA cloning ordered

Olfr10_1_FW	CACCGATAGGAGCCTAGAATGAGCA
Olfr44_1_FW	CACCGACTCCGCAATTGCAAATATG
Olfr52_1_FW	CACCGTTGTGACCATATACAATAGA
ROSA26_1_FW	CACCGGAGTCGTTTTACCCGCCGC
ROSA26_2_FW	CACCGCGATGGAAAATACTCCGAGG

Supplementary Table 5 – Editing-scar sequencing primer

Olfr10_1_FW	CTACACGACGCTCTTCCGATCTNNGGTCTGTGAAGACACAGGAGG
Olfr44_1_FW	CTACACGACGCTCTTCCGATCTNNGCTAGTGAACTTTGTGACAGAGAA
Olfr52_1_FW	CTACACGACGCTCTTCCGATCTNNTGGTATCAGAGTGCTTGCTCCT
ROSA26_1_FW	CTACACGACGCTCTTCCGATCTNNATGGCAAGGGCCAGTTTTCT
ROSA26_2_FW	CTACACGACGCTCTTCCGATCTNNAGCAGGAGAGTATAAAACTCGGG
Olfr10_1_RV	CAGACGTGTGCTCTTCCGATCTGCCTTTCTGCGTGCAGTTAC
Olfr44_1_RV	CAGACGTGTGCTCTTCCGATCTACAGATAGCAACATAGCGGTCA
Olfr52_1_RV	CAGACGTGTGCTCTTCCGATCTGTGTGAATCAATGCCATGAGGAAA
ROSA26_1_RV 1	CAGACGTGTGCTCTTCCGATCTTCAGCGAAAGTAGCTCGC
ROSA26_2_RV	CAGACGTGTGCTCTTCCGATCTAGGATAGGTAGTCATCTGGGGT
VEGFA_3_FW	CTACACGACGCTCTTCCGATCTNNTCGGCCACCACAGGGAAG
MAX_x_FW	CTACACGACGCTCTTCCGATCTNNATTTCCTACGGCCCAGGGAG
COMDA_x_FW	CTACACGACGCTCTTCCGATCTNNTGTCACCACACAGTTACCACC
EMX1_x_FW	CTACACGACGCTCTTCCGATCTNNACCGGAGGACAAAGTACAAACG
HCN1_x_FW	CTACACGACGCTCTTCCGATCTNNAGATCTGACAGAGAAACATTTACCA
MFAP1_x_FW	CTACACGACGCTCTTCCGATCTNNATGTGCTTCAACCCATCACG
FANCF_x_FW	CTACACGACGCTCTTCCGATCTNNTGGCGGGGTCCCAGG
LINC00971_x_FW	CTACACGACGCTCTTCCGATCTNNAGCTGAAATTGGTAAGGTTAGTTTT
SNX1_x_FW 1	CTACACGACGCTCTTCCGATCTNNCAGGCCATTCTCCCTGTGAA
VEGFA_3_RV 1	CAGACGTGTGCTCTTCCGATCTATTGGAATCCTGGAGTGACCC
MAX_x_RV 1	CAGACGTGTGCTCTTCCGATCTAGTGAGGAGGTGGTTCTTGC
COMDA_x_RV	CAGACGTGTGCTCTTCCGATCTATCTAATGTATGGCATGGTGACTA
EMX1_x_RV	CAGACGTGTGCTCTTCCGATCTCTTCGTGGCAATGCGCC
HCN1_x_RV	CAGACGTGTGCTCTTCCGATCTGGCTTATGGCATGGCAAGAC
MFAP1_x_RV	CAGACGTGTGCTCTTCCGATCTCTCATGACTTGGCCTTTGTAGG
FANCF_x_RV	CAGACGTGTGCTCTTCCGATCTGTCTCCAAGGTGAAAGCGGA
LINC00971_x_RV	CAGACGTGTGCTCTTCCGATCTGCAAAGATTGGAGAGATGCGT
SNX1_x_RV	CAGACGTGTGCTCTTCCGATCTAGGATGCCACATGACAGCAA

indexing primer-F-1	AATGATACGGCGACCACCGAGATCTACACCGCTCCACGAACACTCTTTCCCTACACGACGCTCTTCCGATCT
indexing primer-F-2	AATGATACGGCGACCACCGAGATCTACACTATCTTGTAGACACTCTTTCCCTACACGACGCTCTTCCGATCT
indexing primer-F-3	AATGATACGGCGACCACCGAGATCTACACAGCTACTATAACACTCTTTCCCTACACGACGCTCTTCCGATCT
indexing primer-F-4	AATGATACGGCGACCACCGAGATCTACACGCACCAGGCAACACTCTTTCCCTACACGACGCTCTTCCGATCT
indexing primer-F-5	AATGATACGGCGACCACCGAGATCTACACTCAGTACGCAACACTCTTTCCCTACACGACGCTCTTCCGATCT
indexing primer-F-6	AATGATACGGCGACCACCGAGATCTACACCAGAGGATGTACACTCTTTCCCTACACGACGCTCTTCCGATCT
indexing primer-F-7	AATGATACGGCGACCACCGAGATCTACACATTGGTCAACACACTCTTTCCCTACACGACGCTCTTCCGATCT

indexing primer-R-1	CAAGCAGAAGACGGCATACGAGATGAACTGAGCGGTGACTGGAGTTCAGACGTGTGCTCTTCCGATCT
indexing primer-R-2	CAAGCAGAAGACGGCATACGAGATAGGTCAGATAGTGACTGGAGTTCAGACGTGTGCTCTTCCGATCT
indexing primer-R-3	CAAGCAGAAGACGGCATACGAGATCGTCTCATATGTGACTGGAGTTCAGACGTGTGCTCTTCCGATCT
indexing primer-R-4	CAAGCAGAAGACGGCATACGAGATATTCCATAAGGTGACTGGAGTTCAGACGTGTGCTCTTCCGATCT
indexing primer-R-5	CAAGCAGAAGACGGCATACGAGATGACGAGATTAGTGACTGGAGTTCAGACGTGTGCTCTTCCGATCT
indexing primer-R-6	CAAGCAGAAGACGGCATACGAGATAACATCGCGCGTGACTGGAGTTCAGACGTGTGCTCTTCCGATCT
indexing primer-R-7	CAAGCAGAAGACGGCATACGAGATCTAGTGCTCTGTGACTGGAGTTCAGACGTGTGCTCTTCCGATCT
indexing primer-R-8	CAAGCAGAAGACGGCATACGAGATGATCAAGGCAGTGACTGGAGTTCAGACGTGTGCTCTTCCGATCT
indexing primer-R-9	CAAGCAGAAGACGGCATACGAGATTCCATTGCCGGTGACTGGAGTTCAGACGTGTGCTCTTCCGATCT
indexing primer-R10	CAAGCAGAAGACGGCATACGAGATTCGTAAAGCTGTGACTGGAGTTCAGACGTGTGCTCTTCCGATCT
indexing primer-R11	CAAGCAGAAGACGGCATACGAGATCTGACCGCTAGTGACTGGAGTTCAGACGTGTGCTCTTCCGATCT
indexing primer-R12	CAAGCAGAAGACGGCATACGAGATTAGACACTAGGTGACTGGAGTTCAGACGTGTGCTCTTCCGATCT
indexing primer-R13	CAAGCAGAAGACGGCATACGAGATGCATTCTAGCGTGACTGGAGTTCAGACGTGTGCTCTTCCGATCT
indexing primer-R14	CAAGCAGAAGACGGCATACGAGATCCTTGTGAACGTGACTGGAGTTCAGACGTGTGCTCTTCCGATCT

Supplementary Table 6 - Human ON/OFF target sites

**On target:**	**VEGFA**	GGTGAGTGAGTGTGTGCGTGtGG
Off target	MAX	AGTGAGTGAGTGTGTGTGTGgGG

Off target	COMDA	TGTGGGTGAGTGTGTGCGTGaGG
On target	**EMX1**	GAGTCCGAGCAGAAGAAGAAgGG
Off target	HCN1	GAGTTAGAGCAGAAGAAGAAaGG
Off target	MFAP1	GAGTCTAAGCAGAAGAAGAAgAG
On target	FANCF _6	GCTTGAGACCGCCAGAAGCTCGG
Off target	LINC00971	GCTGGAAACCACCAGAAGCTAGG
Off target	SNX1 chr 15	GCCTGAGACTGCCAGAAGCTGGG
