# Supplementary material for: CRISPR-Switch regulates sgRNA activity by Cre recombination for sequential editing of two loci
Source: Nat Commun. 2019 Nov 29;10:5454. doi: 10.1038/s41467-019-13403-y (PMC6884486; doi:10.1038/s41467-019-13403-y)
Supplement: Supplementary file 2 — Reporting Summary [file 41467_2019_13403_MOESM2_ESM.pdf]

nature research

Corresponding author(s): Ulrich Elling

Last updated by author(s): Oct 31, 2019

Reporting Summary

Nature Research wishes to improve the reproducibility of the work that we publish. This form provides structure for consistency and transparency in reporting. For further information on Nature Research policies, see Authors & Referees and the Editorial Policy Checklist.

Statistics

For all statistical analyses, confirm that the following items are present in the figure legend, table legend, main text, or Methods section.

n/a	Confirmed	
☐	☒	The exact sample size (*n*) for each experimental group/condition, given as a discrete number and unit of measurement
☐	☒	A statement on whether measurements were taken from distinct samples or whether the same sample was measured repeatedly
☐	☒	The statistical test(s) used AND whether they are one- or two-sided<br>*Only common tests should be described solely by name; describe more complex techniques in the Methods section.*
☐	☒	A description of all covariates tested
☐	☒	A description of any assumptions or corrections, such as tests of normality and adjustment for multiple comparisons
☐	☒	A full description of the statistical parameters including central tendency (e.g. means) or other basic estimates (e.g. regression coefficient) AND variation (e.g. standard deviation) or associated estimates of uncertainty (e.g. confidence intervals)
☐	☒	For null hypothesis testing, the test statistic (e.g. *F*, *t*, *r*) with confidence intervals, effect sizes, degrees of freedom and *P* value noted<br>*Give P values as exact values whenever suitable.*
☐	☒	For Bayesian analysis, information on the choice of priors and Markov chain Monte Carlo settings
☐	☒	For hierarchical and complex designs, identification of the appropriate level for tests and full reporting of outcomes
☐	☒	Estimates of effect sizes (e.g. Cohen's *d*, Pearson's *r*), indicating how they were calculated

Our web collection on statistics for biologists contains articles on many of the points above.

Software and code

Policy information about availability of computer code

Data collection	BD FACSDiva and BD Accuri C6 software was used for data collection using flow cytometry. The Illumina platform MySeq was used for next generation sequencing.
Data analysis	FlowJo X (flow cytometry), GraphPad Prism 6 (graphs preparation), custom code is available at https://github.com/GMichlits/CRISPR-Switch.

For manuscripts utilizing custom algorithms or software that are central to the research but not yet described in published literature, software must be made available to editors/reviewers. We strongly encourage code deposition in a community repository (e.g. GitHub). See the Nature Research guidelines for submitting code & software for further information.

Data

Policy information about availability of data

All manuscripts must include a data availability statement. This statement should provide the following information, where applicable:
- Accession codes, unique identifiers, or web links for publicly available datasets
- A list of figures that have associated raw data
- A description of any restrictions on data availability

The data supporting the findings of this study are available within the paper and its supplementary information files. All materials are available upon request. Requests should be addressed to UE (ulrich.elling@imba.oeaw.ac.at). Code is available at https://github.com/GMichlits/CRISPR-Switch.

Field-specific reporting

Please select the one below that is the best fit for your research. If you are not sure, read the appropriate sections before making your selection.

☒ Life sciences ☐ Behavioural & social sciences ☐ Ecological, evolutionary & environmental sciences

For a reference copy of the document with all sections, see nature.com/documents/nr-reporting-summary-flat.pdf

Life sciences study design

All studies must disclose on these points even when the disclosure is negative.

Sample size	Sample size was based on experience and that our proof-of-concept in vivo CRISPR experiment is based on a genetic mouse model fact that previous published data (DOI 10.1016/j.ccr.2005.07.004) show that only 6% NF1 only mutant mice show gliomas whereas 100% of p53; NF1 compound mutant mice show glioma development. As such 14 or more mice per experimental arm are sufficient to reach statistical significance.
Data exclusions	N/A
Replication	Different litter of mice were infected with virus targeting the same gene on different days (biological replicates). 2 different sgRNAs were used for each gene.
Randomization	Pups of the same litter were randomized and received received sgRNA-harboring lentivirus.
Blinding	Data collection, staining as well as histological analysis was done by 3 different blinded investigators.

Reporting for specific materials, systems and methods

We require information from authors about some types of materials, experimental systems and methods used in many studies. Here, indicate whether each material, system or method listed is relevant to your study. If you are not sure if a list item applies to your research, read the appropriate section before selecting a response.

Materials & experimental systems

n/a	Involved in the study	
☐	☒	Antibodies
☐	☒	Eukaryotic cell lines
☒	☐	Palaeontology
☐	☒	Animals and other organisms
☒	☐	Human research participants
☒	☐	Clinical data

Methods

n/a	Involved in the study	
☒	☐	ChIP-seq
☐	☒	Flow cytometry
☒	☐	MRI-based neuroimaging

Antibodies

Antibodies used	PE anti-mouse/human CD15 (SSEA-1) clone MC-480, Biolegend<br>Anti-Mouse CD16/CD32 Clone 93, eBioscience (Fc gamma block)<br>p53 (Leica NCL-L-p53-CM5p) and ki67 (Abcam ab15580). Goat anti-rabbit secondary (Vector Labs BA-1000) was applied for 30 minutes followed by ABC regent (Vector Labs PK-6100) for 25 minutes and developed with DAB (Vector Labs SK-4100) for 4 minutes or less.<br>APC anti-human CD81, clone 5A6, Biolegend #349510
Validation	All antibodies are commercially available and validated by the manufacturers. Signal loss in deletion experiments as shown in the manuscript further confirms functionality and specificity of the antibodies used.

Eukaryotic cell lines

Policy information about cell lines

Cell line source(s)	AN3-12 cells are a clonal derivative of HMSc2, mouse embryonic stem cells derived in the laboratory (Elling et al. 2011 and 2017); NIH3T3 cells were purchased from ATCC, PlatinumE cells were purchased from Cell Biolabs, and LentiX cells were purchased from Clontech. A375 cells were obtained from Cancer Cell Line Encyclopedia. Hap1 cells were obtained from Haplogen and Hela cells from ATCC.
Authentication	Hap1 cells, Hela cells, and A375 cells were authenticated by SNP profiling.

Mycoplasma contamination	All cells lines routinely tested negative for mycoplasma contamination.
Commonly misidentified lines (See ICLAC register)	Cells used in the study are not commonly misidentified cell lines.

Animals and other organisms

Policy information about studies involving animals; ARRIVE guidelines recommended for reporting animal research

Laboratory animals	Tg(Nes-cre/ERT2)1Adra; Gt(ROSA)26Sortm1.1(CAG-cas9*,-EGFP)Fezh; male and female mice; 0-10 month of age
Wild animals	N/A
Field-collected samples	N/A
Ethics oversight	Animal husbandry, ethical handling of mice and all animal work were carried out according to guidelines approved by Canadian Council on Animal Care and under protocols approved by the Centre for Phenogenomics Animal Care Committee (18-0272H).

Note that full information on the approval of the study protocol must also be provided in the manuscript.

Flow Cytometry

Plots

Confirm that:

☒ The axis labels state the marker and fluorochrome used (e.g. CD4-FITC).

☒ The axis scales are clearly visible. Include numbers along axes only for bottom left plot of group (a 'group' is an analysis of identical markers).

☒ All plots are contour plots with outliers or pseudocolor plots.

☒ A numerical value for number of cells or percentage (with statistics) is provided.

Methodology

Sample preparation	Cells were cultured as described in Material and Method section. For Flow cytometry, cell were detached and singularized using standard techniques and resuspended in media for measurement. When needed, antibody staining was performed as described in detail in Materials and Methods section.
Instrument	BD LSR Fortessa, BD FACSARIA III, BD Accuri C6
Software	BD FACSDiva and BD Accuri C6 software (data collection), FlowJo X (data analysis)
Cell population abundance	Sorting was repeated until sample was > 99.5 % pure.
Gating strategy	FSC/SSC was used to select initial cell population. Ratio of FSC-H or FSC-W to FSC-A or SSC-H or SSC-W to SSC-A was used to select single cell population. Details and example gatings, including examples of positive and negative populations are shown in Supplementary Table 14

☒ Tick this box to confirm that a figure exemplifying the gating strategy is provided in the Supplementary Information.
